# A Multi‐Faceted Approach to Explore the Role of Inflammatory CAFs, Providing Prognostic Value and Therapeutic Implications in Lung Adenocarcinoma

**DOI:** 10.1155/ijog/6004629

**Published:** 2025-12-12

**Authors:** Xiaoyue Zhou, Cong Fu, Ying Fu, Ting Jiao, Chenyu Zhao, Dan Yang

**Affiliations:** ^1^ Department of Oncology, Changzhou Cancer (Fourth People’s) Hospital, Changzhou, China; ^2^ School of Pharmacy, Queen’s University Belfast, Belfast, UK, qub.ac.uk

**Keywords:** inflammatory cancer-associated fibroblasts, lung adenocarcinoma, machine learning, prognosis, single-cell RNA-sequencing

## Abstract

**Background:**

Lung adenocarcinoma (LUAD) is the most common subtype of lung cancer, where its complex tumor microenvironment (TME) significantly influences disease progression and treatment response. Inflammatory cancer‐associated fibroblasts (iCAFs), as a key component of the TME, can promote tumor immune evasion and drug resistance. However, the characteristics of iCAFs in LUAD and their clinical significance have not been fully elucidated.

**Methods:**

The bulk RNA data and scRNA‐seq data of LUAD from public databases were integrated to identify and characterize iCAFs subsets and screen their feature genes. iCAF‐based signature (ICAFBS) was generated using multiple machine learning algorithms and confirmed in multiple independent cohorts. The relationship between ICAFBS and immune landscape as well as drug sensitivity was further analyzed. Finally, a series of functional studies were conducted to elucidate the role of PFN2 in LUAD cell lines.

**Results:**

The iCAF subtype identified by single‐cell analysis was enriched in LUAD and closely linked to poor prognosis. Based on 145 iCAF characteristic genes, ICAFBS was finally screened out four key genes, MGP, LOXL2, FSTL3, and PFN2. ICAFBS demonstrated excellent prognostic predictive capabilities and was validated in multiple external datasets. Patients in the high ICAFBS group showed significant high expression of multiple immune checkpoints. Notably, silencing PFN2 inhibited cell viability and proliferation in LUAD cells, highlighting its potential as a therapeutic target.

**Conclusion:**

As a novel prognostic signature, ICAFBS can effectively predict the clinical outcome, immune landscape and treatment response of patients, providing an important reference for the individualized treatment of LUAD.

## 1. Introduction

Lung adenocarcinoma (LUAD) represents the predominant histological subtype of lung cancer, constituting nearly 40% of all LC cases worldwide [1–3]. Advances in early detection, targeted therapies, and immunotherapies have been achieved, however, the outcome of LUAD cases remains poor since late diagnosis, high heterogeneity, and treatment resistance [4–6]. The 5‐year survival rate for advanced LUAD is still less than 20% [7–9], underscoring the urgent need for novel prognostic biomarkers and therapeutic targets to improve patient outcomes [10]. Given the complexity of the biological behavior of LUAD, a deeper understanding of the contribution of the tumor microenvironment (TME) in its progression is critical for the design of personalized treatment strategies [11].

Accumulating research has demonstrated inflammatory‐mediated microenvironment exerts an essential effect on tumorigenesis, metastasis, and treatment failure in various cancers, including LUAD [12, 13]. Chronic inflammation not only promotes genetic mutations and genomic instability but also facilitates angiogenesis, tissue remodeling, and immune evasion [14, 15]. In LUAD, inflammatory signaling pathways, such as IL‐6/STAT3, NF‐*κ*B, and TGF‐*β*, are frequently activated, fostering a pro‐tumorigenic environment [16–18]. Moreover, inflammation‐driven recruitment of immunosuppressive cells, including regulatory T cells (Tregs), tumor‐associated macrophages (TAMs), and myeloid‐derived suppressor cells (MDSCs) [19], further enhances immune escape mechanisms [20–22]. Recent studies have highlighted that the interplay between cancer cells and inflammatory components of the TME can dictate responses to immunotherapy and contribute to drug resistance [23, 24], emphasizing the importance of deciphering the inflammatory landscape in LUAD.

Among the diverse stromal components of the TME, cancer‐associated fibroblasts (CAFs) have emerged as key players in modulating tumor behavior [25, 26]. Notably, inflammatory CAFs (iCAFs) represent a distinct subset of CAFs characterized by the secretion of pro‐inflammatory cytokines and chemokines, such as CXCL14, CXCL12, and CCL2. These cells actively participate in shaping an immunosuppressive and pro‐tumorigenic microenvironment through remodeling of the extracellular matrix (ECM), promotion of angiogenesis, and modulation of immunocyte infiltration [27, 28]. Emerging evidence suggests that iCAFs were closely associated with worse outcomes and facilitated tumor growth and progression [29, 30]. Despite the recognized importance of iCAFs in LUAD, comprehensive transcriptomic profiling remains scarce. Moreover, only a limited number of studies have attempted to construct prognostic models based on iCAF‐related gene signatures. Furthermore, the interplay between iCAFs and immune responses, particularly in the context of immunotherapy, has not been systematically elucidated.

In this study, we identified iCAFs by deciphering LUAD heterogeneity and subsequently derived feature genes for iCAFs to develop prognostic models (ICAFBS) elucidating their relationship with LUAD progression. Then, we demonstrated the robust predictive performance of ICAFBS for clinical outcomes in LUAD patients and explored its association with the immune landscape. In addition, multiple functional assays supported the pro‐tumorigenic role of PFN2 in LUAD, suggesting that it may serve as a promising therapeutic target.

## 2. Materials and Methods

### 2.1. Data Collection

RNA‐seq data and corresponding clinical data for LUAD cases were collected from The Cancer Genome Atlas (TCGA). Additionally, gene expression profiles and clinical information for four other LUAD datasets (GSE31210, GSE50081, GSE68465, and GSE72094) were collected from the Gene Expression Omnibus (GEO) database.

The LUAD single‐cell RNA sequencing (scRNA‐seq) dataset (GSE131907) were downloaded from the GEO database [31]. The spatial transcriptomics data of LUAD (E‐MTAB‐13530) was obtained from EMBL‐EBI database.

### 2.2. Single‐Cell RNA‐Seq Analysis

We processed scRNA‐seq data through the Seurat package. Initial quality control involved filtering out cells exhibiting over 10% mitochondrial gene expression. Subsequently, we identified 2000 highly variable genes for downstream analysis. Harmony analysis was applied to mitigate batch effects. Cell clustering was achieved using FindClusters and FindNeighbors, followed by visualization via the UMAP algorithm. Cell types were annotated according to their characteristic marker genes.

For differential gene expression (DEG) analysis, the FindMarkers function within Seurat was employed. Additionally, CellChat was utilized to explore intercellular communication networks. To explore functional enrichment of different cell subtypes, we incorporated the ClusterGVis and org.Hs.eg.db R packages. Gene set variation analysis (GSVA) was performed using the R package GSVA. Differential pathway enrichment between groups was assessed with the limma package, and multiple‐testing correction was applied using the Benjamini–Hochberg method. Pathways with a false discovery rate (FDR) < 0.05 were considered statistically significant.

### 2.3. Development of iCAF‐Based Signature (ICAFBS)

To build a reliable prognostic model with strong predictive capability, we implemented the following procedures:

The model development pipeline began with the identification of prognostic ICAFBS in the TCGA‐LUAD (training cohort) via univariate Cox regression. Subsequently, the 88 algorithm combinations were applied to these selected genes to build candidate models, which were trained and internally validated using leave‐one‐out cross‐validation (LOOCV) [31]. To assess their generalizability, all models underwent rigorous external validation on three independent GEO cohorts (GSE31210, GSE50081, GSE68465, and GSE72094). Finally, model performance was quantified using Harrell’s concordance index (C‐index), and the model achieving the highest average C‐index across all validation datasets was selected as the optimal signature. A complete list of algorithms and further methodological details are provided in the Supplementary Methods and Supplementary Table 1.

### 2.4. Immune Landscape Analysis

To determine the relationship between ICAFBS and immunocyte infiltration in LUAD, we quantified the infiltration of immunocytes using various algorithms including XCell, CIBERSORT, TIMER, QUANTISEQ, MCPCOUNTER, EPIC, and CIBERSORT.

### 2.5. Immune Function Analysis

Single‐sample gene set enrichment analysis (ssGSEA) is a commonly applied method for estimating the enrichment level of specific gene sets in individual samples. The ssGSEA score indicates the extent to which a gene set is consistently activated or suppressed within a given sample. To explore immune function activity among two risk groups, we computed GSVA scores across 13 gene sets [31]. Subsequently, immune function pathways exhibiting significant differences between two risk groups were identified using the limma package.

### 2.6. Immunophenoscore (IPS) Analysis

To assess immunogenicity involving immunomodulators, immunosuppressive cells, MHC molecules, and effector cells, we applied the Immunophenoscore (IPS) algorithm. This algorithm estimates IPS scores based on representative cell‐type gene expression profiles using machine learning techniques. Notably, higher IPS scores reflect improved responsiveness to immunotherapy. IPS data for LUAD patient samples were retrieved from The Cancer Immunome Atlas (TCIA) (https://tcia.at/home).

### 2.7. Quantitative Real‐Time Polymerase Chain Reaction (qRT‐PCR)

Total RNA was isolated from two LUAD cell lines (A549 and H1975) and a normal lung epithelial cell line (BEAS‐2B) [32] using RNA‐easy Isolation Reagent (Vazyme Biotech, Nanjing, China). The extracted RNA was then reverse transcribed into cDNA with the PrimeScript RT reagent kit [33] (Takara, DRR037A). Quantitative real‐time PCR (qRT‐PCR) was performed on a Bio‐Rad CFX96 system (Bio‐Rad, Hercules, California, United States). Gene expression levels were calculated using the comparative Ct method and normalized to GAPDH as the internal control. The knockdown sequences of PFN2 are provided in Supplementary Table 2. Primer sequences for the target and reference genes are provided in Supplementary Table 3.

### 2.8. Cell Counting Kit‐8 (CCK‐8) Assay

Cell proliferation was evaluated using the Cell Counting Kit‐8 (CCK‐8; Bioscience Technology Co., Ltd.) according to the manufacturer’s instructions. Cells were seeded into 96‐well plates at a density of 2000 cells per well. After allowing the cells to adhere (0 h), absorbance was measured at 0, 24, 48, 72, and 96 h. At each time point, the culture medium was carefully aspirated, and the wells were gently rinsed with phosphate‐buffered saline (PBS). Subsequently, 100 *μ*L of CCK‐8 working solution—prepared by diluting the CCK‐8 reagent 1:10 in serum‐free medium—was added to each well. After a 2‐h incubation at 37°C, the absorbance was recorded at 450 nm using a multifunctional microplate reader [31].

### 2.9. EdU Assay

Cells were seeded in 96‐well plates at a density of 5000 cells per well and cultured in DMEM supplemented with 10% FBS for 24 h. After incubation with 50 *μ*M EdU solution for 2 h, cells were fixed with 4% paraformaldehyde and permeabilized using 0.5% Triton X‐100. EdU incorporation was detected by staining with 1× Apollo reaction reagent for 30 min, followed by nuclear counterstaining with DAPI. Fluorescence images were captured using a fluorescence microscope (Carl Zeiss Microscopy, Germany) [31].

### 2.10. Colony Formation Assay

Cells were seeded into 6‐well plates at a density of 300 cells per well and cultured under standard conditions for 10 days until visible colonies formed. Colonies were then fixed with 4% paraformaldehyde for 20 min and stained with 0.1% crystal violet for 30 min at room temperature. The plates were washed, air‐dried, and colonies containing more than 50 cells were counted under a microscope [34].

### 2.11. Statistical Analysis

All bioinformatics analyses were performed in R (version 4.3.3). Kaplan–Meier survival analysis and the log‐rank test (via the “survival” package) were used to compare overall survival (OS) across subgroups. Univariate and multivariate Cox regression identified independent prognostic factors. Model performance was assessed through ROC curve analysis and AUC calculation using “timeROC” [35]. qRT‐PCR data were analyzed with Student’s *t* test. Unless specified otherwise, statistical significance was set at *p* < 0.05.

## 3. Results

### 3.1. Single‐Cell Transcriptomic Atlas of LUAD Samples

**Figure 1:**
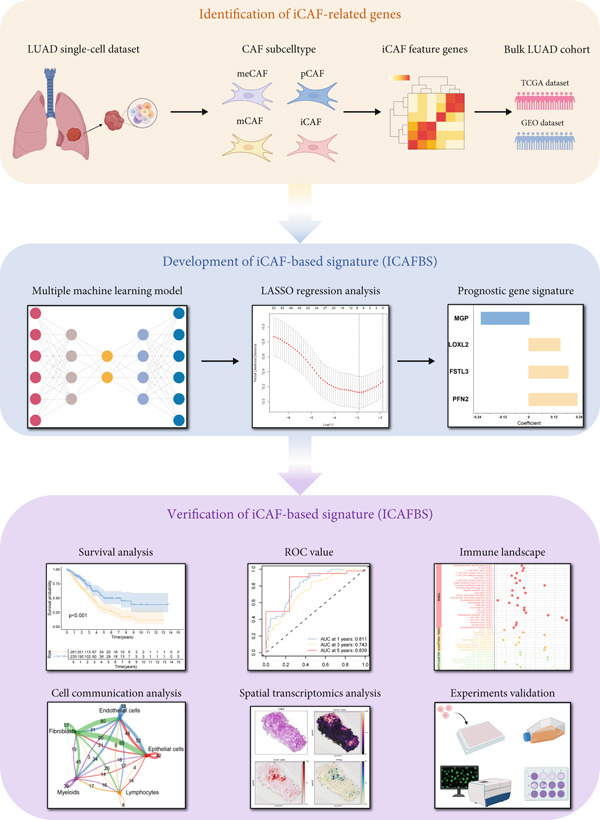
Workflow of the present study.

Figure 1 provides an overview of the study design.

To decipher LUAD heterogeneity, we first performed a LUAD scRNA‐seq dataset (GSE131907) from the GEO database. Following quality control, which excluded potential cell doublets, we retained 27,356 cells for subsequent analysis. To remove batch effects, we employed the Harmony algorithm to address these two datasets. We further used the Harmony‐corrected principal components to generate a unified UMAP. Figure 2a demonstrates the correlation between all cells and clinicopathological types. After performing clustering analysis, a total of 11 clusters were obtained in LUAD (Figure 2b). These clusters were categorized into five major cell types using classical cell markers (Figure 2c). The distribution of different cell types in the overall cell population was shown in Figure 2d. Next dot plot demonstrated the specific cells markers of each cell type (Figure 2e). Additionally, the differences in the proportions of these five cell types were compared in pathological subgroups, further reflecting the heterogeneity of LUAD (Figure 2f).

Figure 2Single‐cell transcriptomic atlas of LUAD samples. (a) UMAP plot of all cells colored by pathological group. (b) UMAP plot showing different identified clusters. (c) UMAP visualization of five major cell types. (d) Distribution of the five cell types and corresponding cell markers. (e) Dot plot of feature genes of each cell type. (f) Proportions of different cell types in terms of pathological group.

**Figure:**
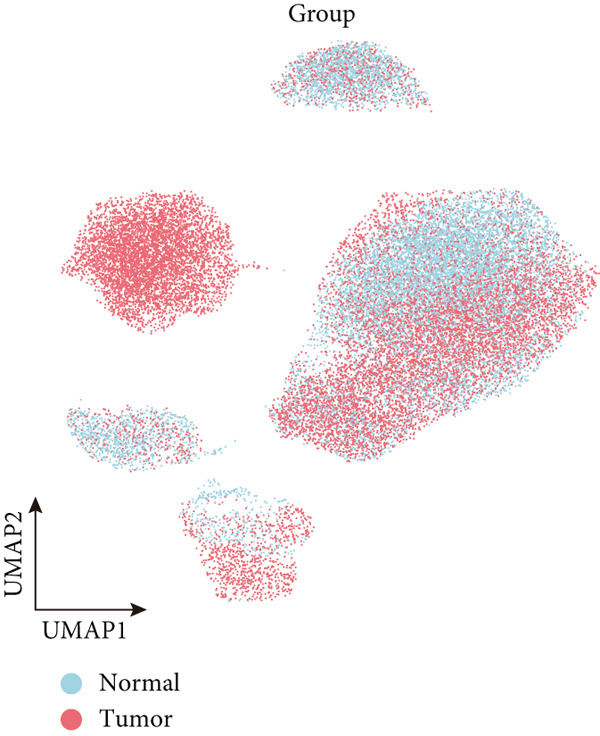
(a)

**Figure:**
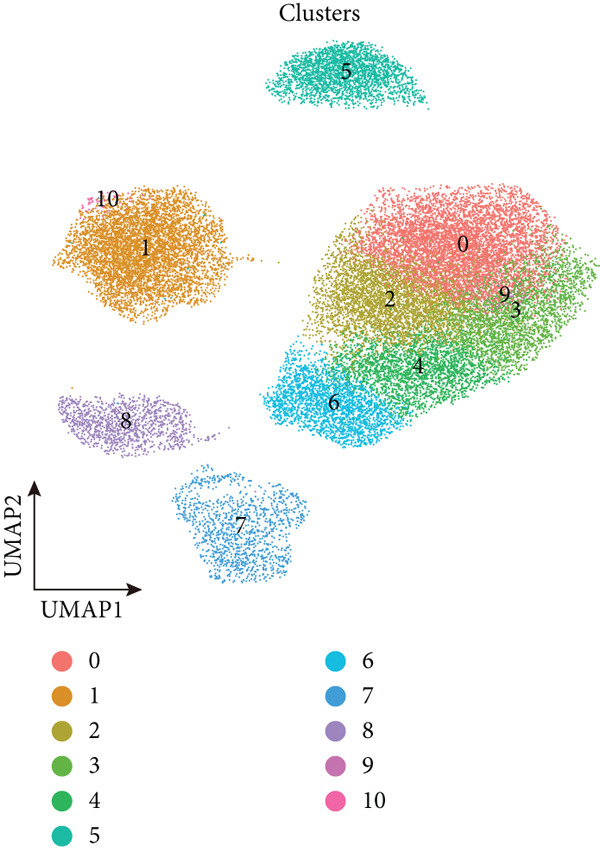
(b)

**Figure:**
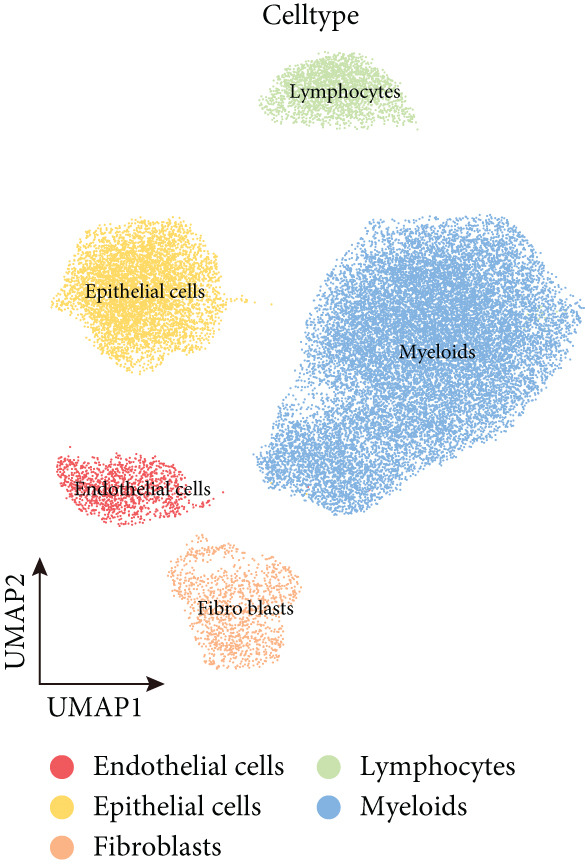
(c)

**Figure:**
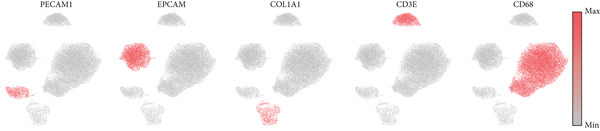
(d)

**Figure:**
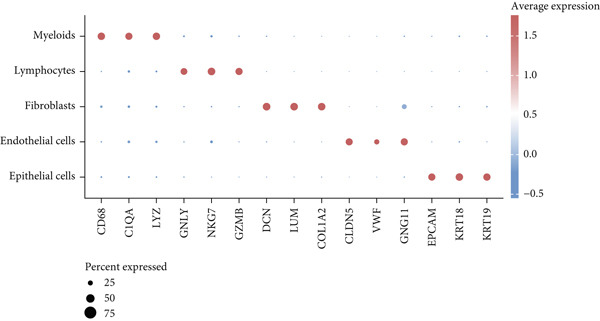
(e)

**Figure:**
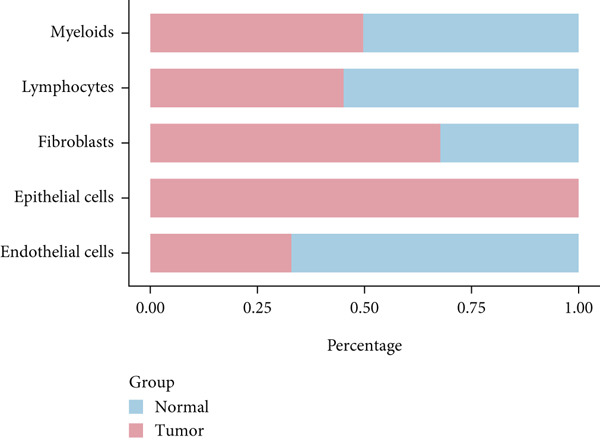
(f)

### 3.2. Inflammatory CAFs (iCAFs) Are Associated With LUAD Development

Higher percentage of fibroblasts (FBs) in tumor group disclosed that FBs may serve as a player in LUAD progression. Therefore, we extracted FBs populations from tumor group and renamed them as CAFs. Then, cluster analysis obtained a total of six clusters (Figure 3a). According to the specific markers mentioned in a previous report [36], we identified four different subtypes of CAFs, including iCAFs, matrix CAF (mCAFs), metabolic CAF (meCAFs), as well as proliferative CAF (pCAFs) (Figure 3b). The corresponding cell markers for each CAFs subtype were demonstrated in Figure 3c and Supplementary Table 4. Based on the differences in the CAF subtype proportions between normal and tumor group, we observed that iCAFs and mCAFs were more predominant in the tumor group (Figure 3d). Subsequently, we verified the functions involved in the four subtypes by enrichment analysis. Consistent with previous studies, iCAFs was associated with inflammation response, immune system regulation, and oxidative stress, whereas mCAFs was correlated with extracellular matrix organization as well as cell migration (Figure 3e). Performing deconvolution algorithm on TCGA‐LUAD bulk data, the prognostic value of the four subtypes in LUAD was detected. Cox regression unearthed that only iCAFs was significantly associated with OS in LUAD (Figure 3f). Survival analysis demonstrated that high infiltration levels of iCAFs were strongly associated with poor clinical outcomes (Figure 3g). Subsequently, we obtained a total of 145 iCAFs feature genes (IFGs) by differential expression analysis (Figure 3h). Further GSVA analysis uncovered that IFGs were significantly enriched in functions such as immune system processes, complement activation, and inflammatory response (Figure 3i).

Figure 3Inflammatory CAFs (iCAFs) are associated with LUAD development. (a) UMAP visualization of different identified clusters. (b) UMAP plot showing diverse subtypes of CAFs. (c) Differential expression analysis demonstrates the feature genes for each subtype. (d) Proportions of four subtypes of CAFs in terms of pathological group. (e) Function enrichment of each CAF subtype. (f) Multivariate cox of each CAF subtype for the OS in LUAD. (g) Survival curves demonstrating iCAFs are dramatically associated with poor patient prognosis (h) Volcano plot showing differentially expressed genes (DEGs) of iCAFs. (i) Function enrichment of DEGs of iCAFs.

**Figure:**
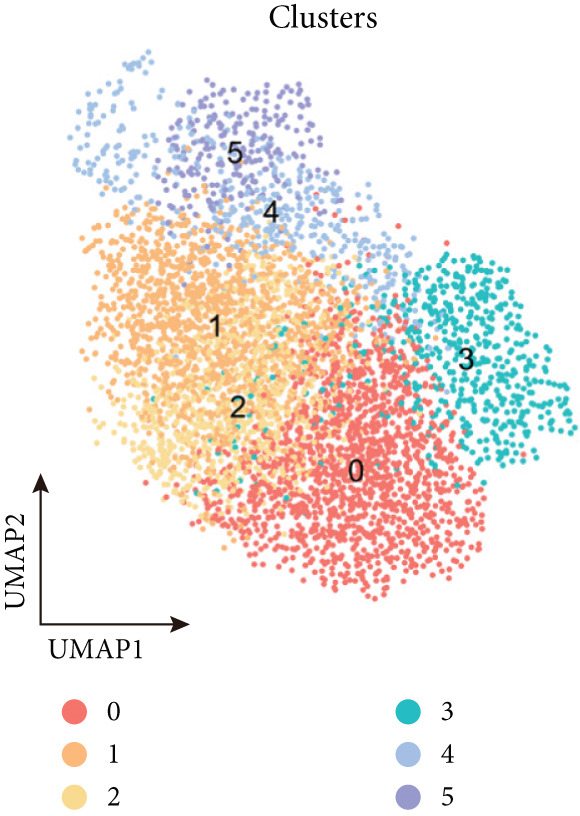
(a)

**Figure:**
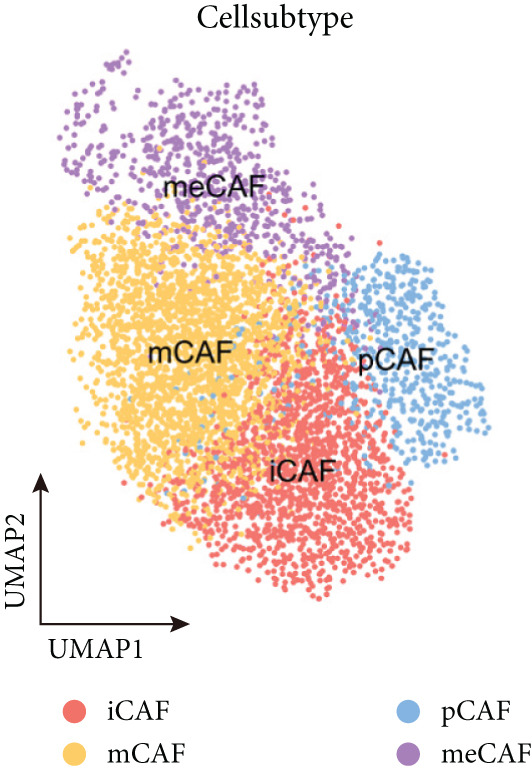
(b)

**Figure:**
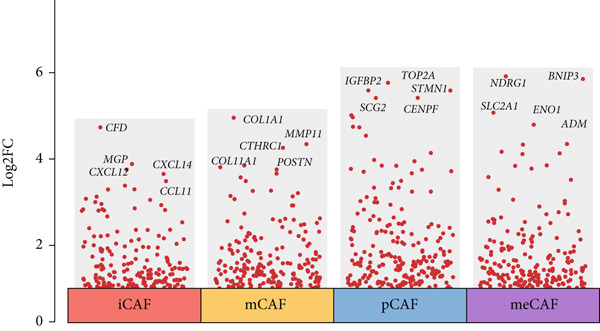
(c)

**Figure:**
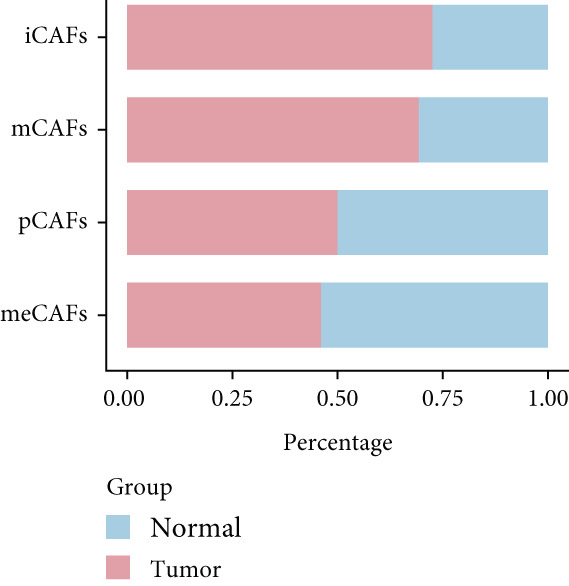
(d)

**Figure:**
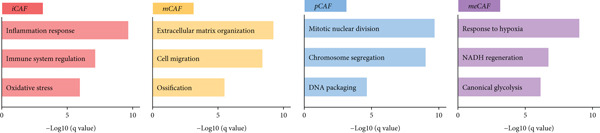
(e)

**Figure:**
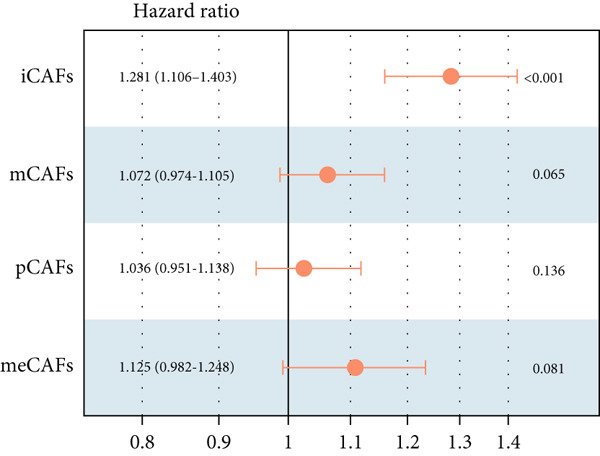
(f)

**Figure:**
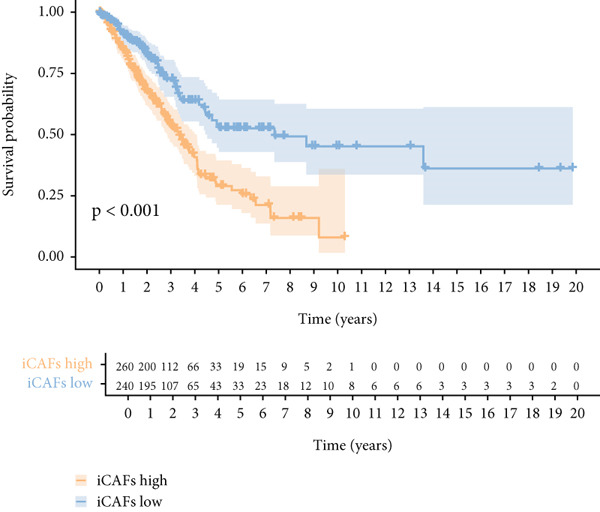
(g)

**Figure:**
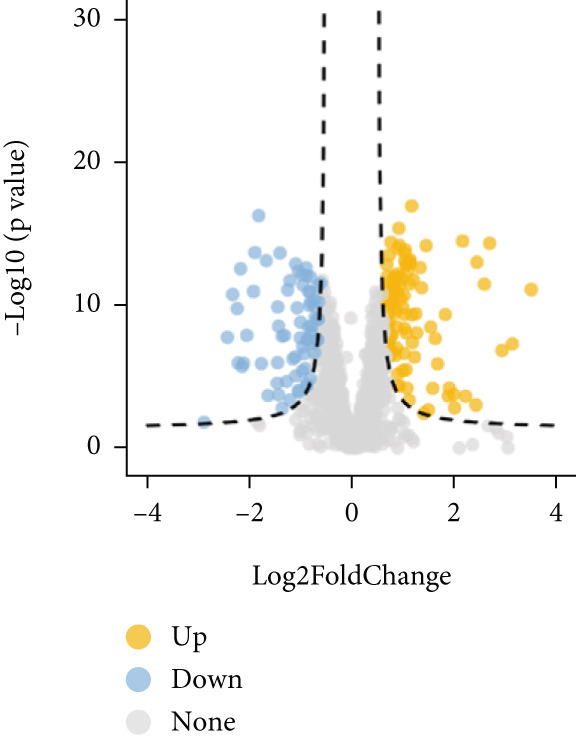
(h)

**Figure:**
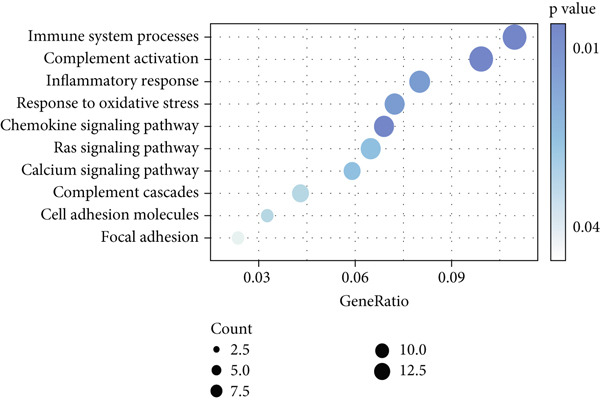
(i)

### 3.3. Construction of iCAF‐Based Signature (ICAFBS) by Using Integrated Machine Learning

To further elucidate the critical role of iCAFs in LUAD prognosis, we developed the ICAFBS based on 145 IFGs of iCAFs. First, TCGA‐LUAD cohort was selected as a training set, and GSE42127, GSE50081, GSE68465 as well as GSE72094 were used for validation. Univariate cox was applied to determine 56 candidate features with remarkable prognostic value (Supplementary Table 5). Subsequently, we employed 88 algorithmic patterns from 10 combinations of machine learning algorithms to the above 56 genes. Based on the ranking of average C‐index, we chose the algorithmic pattern (lasso + stepcox[forward]) with the highest AUC index (0.81) to further identify prominent prognostic features (Figure 4a). The optimal *λ*‐value was generated through LASSO analysis, which resulted in 11 prognostic genes (Figure 4b). Finally, four key genes (MGP, LOXL2, FSTL3, and PFN2) were determined for the model construction using multivariate regression (Supplementary Table 6). The correlation coefficients of each feature in the model were shown in Figure 4c. Based on the median risk score, patients were divided into high‐risk and low‐risk groups [37]. In the TCGA‐LUAD cohort, a greatly worse outcome was observed in patients with high ICAFBS score (Figure 4d). ROC analyses disclosed that the AUC value of ICAFBS was higher than 0.7 at 1‐, 3‐, and 5‐year intervals in the TCGA set, demonstrating strong predictive ability (Figure 4i). Moreover, similar results were found in the other four validation sets (Figures 4e, 4f, 4g, 4h, 4i, 4j, 4k, 4l, and 4m).

Figure 4Construction of iCAF‐based signature (ICAFBS) by using integrated machine learning (a) A total of 88 candidate models were constructed using a tenfold cross‐validation framework, and their C‐index values were estimated across LUAD cohorts. (b) The optimal *λ* value obtained from the LASSO analysis was applied to reduce model overfitting. (c) Regression coefficients of each gene generated in stepwise Cox. (d–h) Survival analysis of OS based on ICAFBS in different datasets, including (d) TCGA‐LUAD, (e) GSE42127, (f) GSE50081, (g) GSE68465, and (h) GSE72094. (i–m) ROC curves of ICAFBS in different LUAD cohorts.

**Figure:**
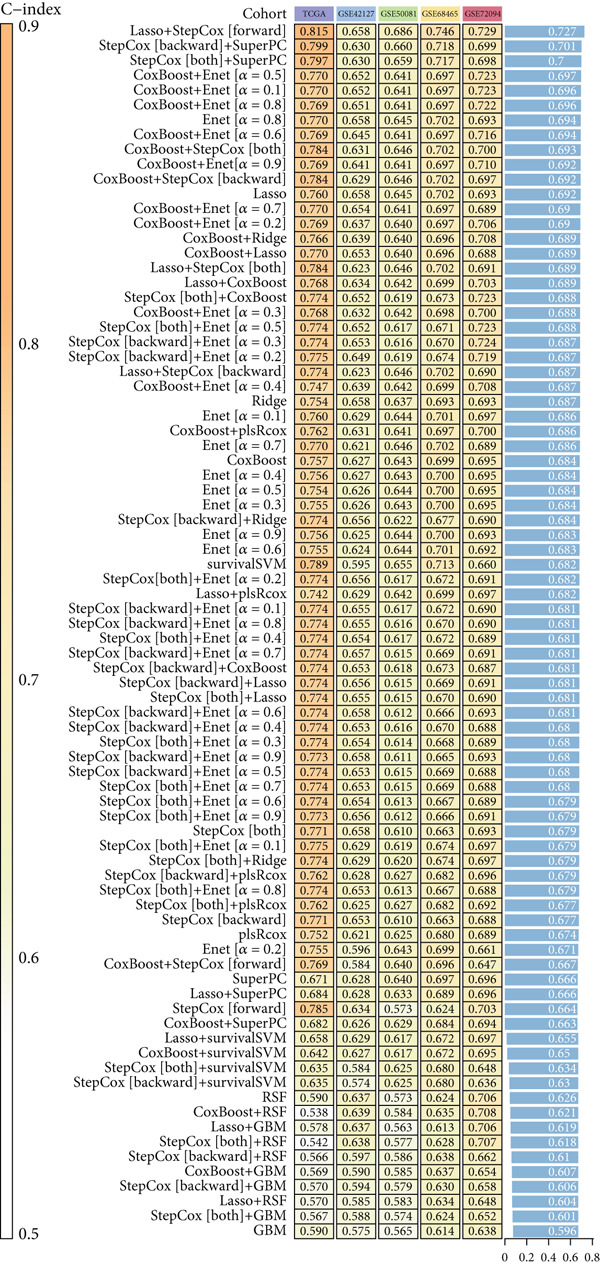
(a)

**Figure:**
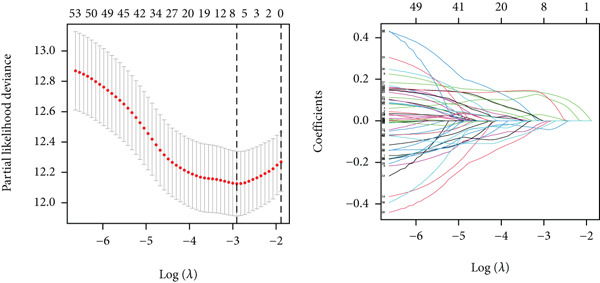
(b)

**Figure:**
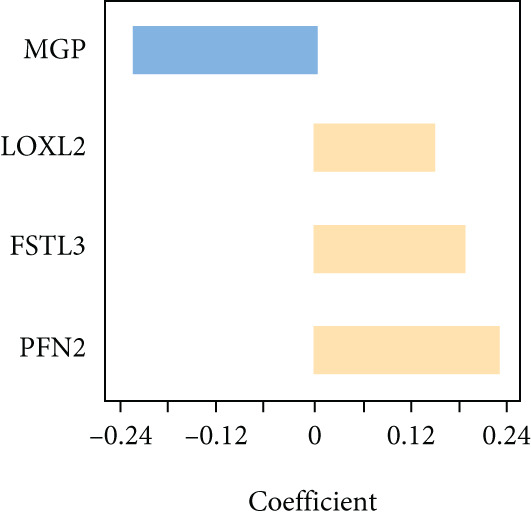
(c)

**Figure:**
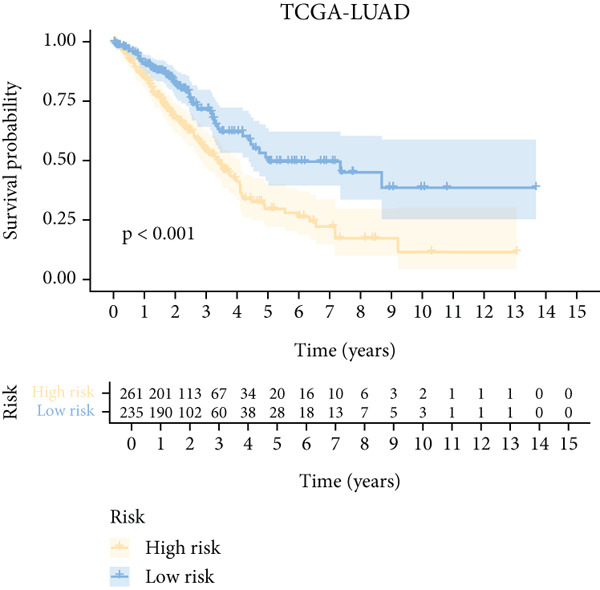
(d)

**Figure:**
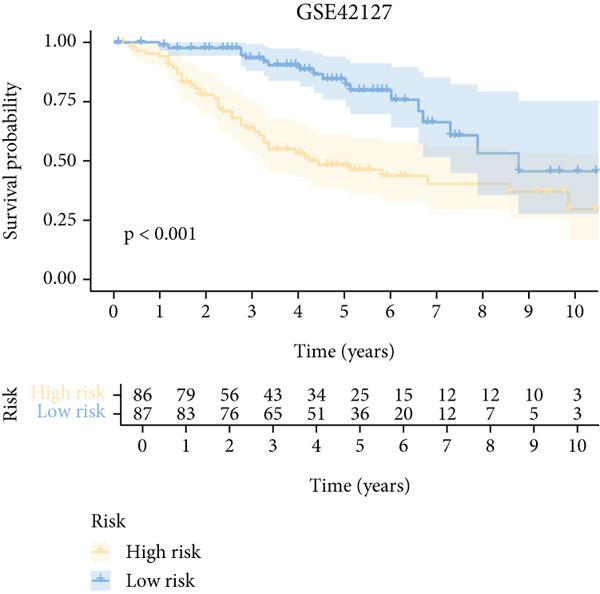
(e)

**Figure:**
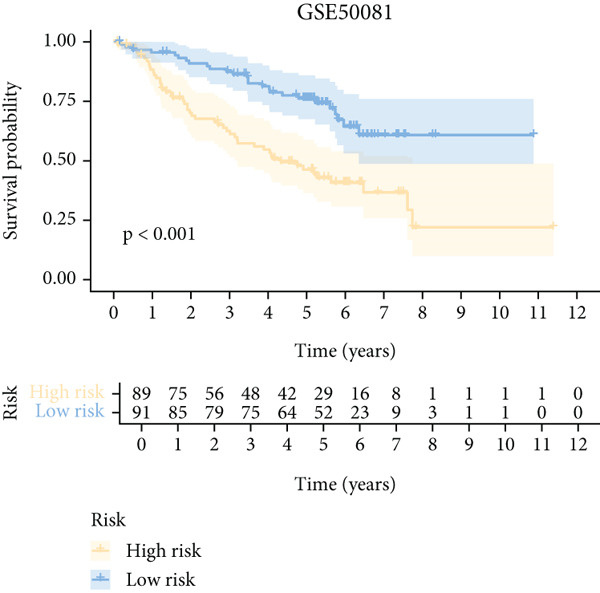
(f)

**Figure:**
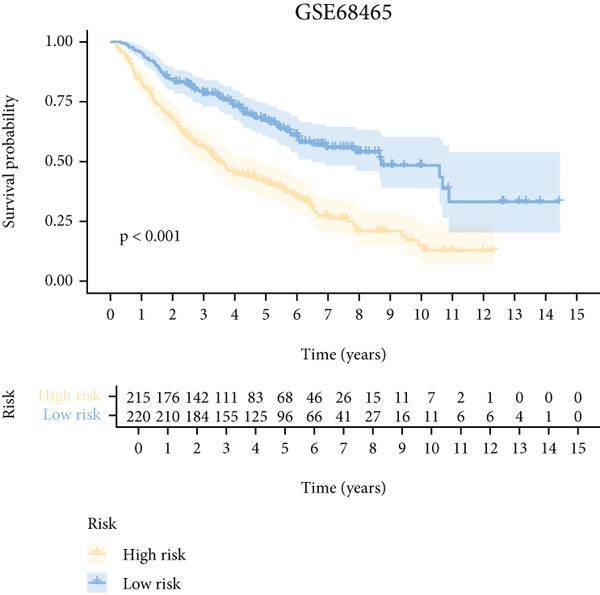
(g)

**Figure:**
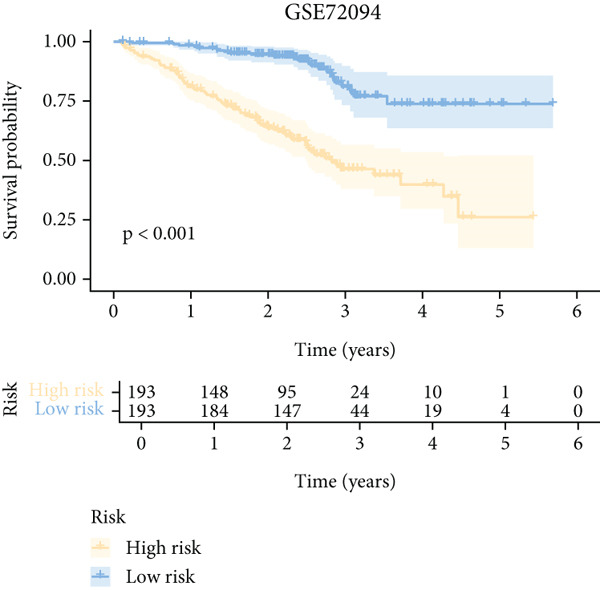
(h)

**Figure:**
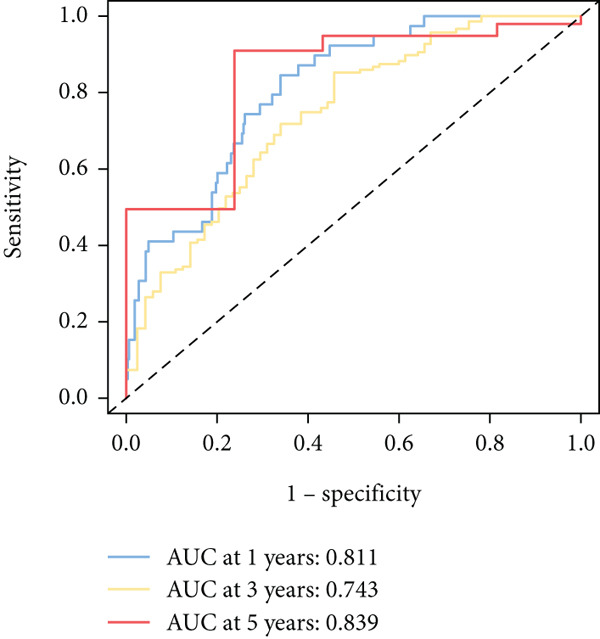
(i)

**Figure:**
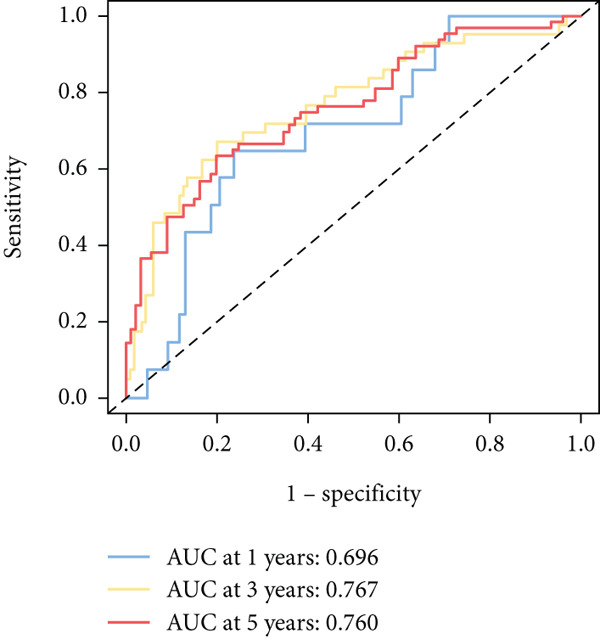
(j)

**Figure:**
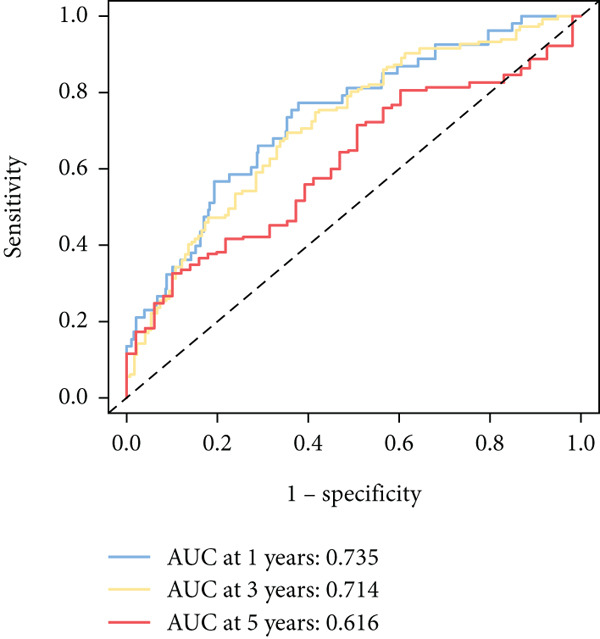
(k)

**Figure:**
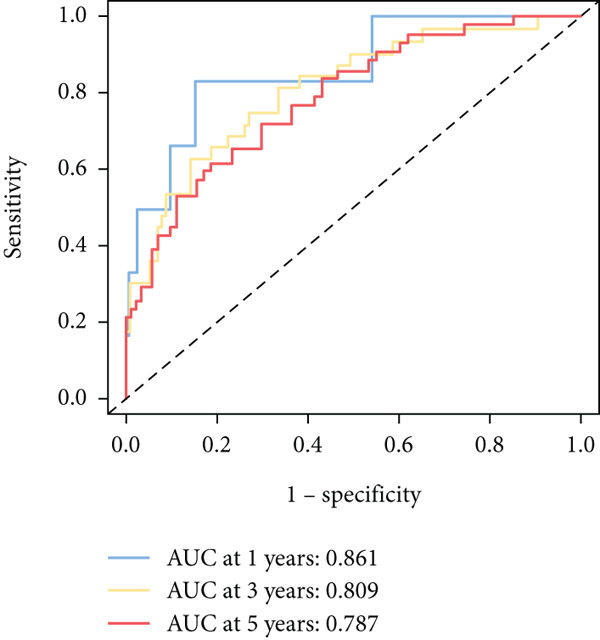
(l)

**Figure:**
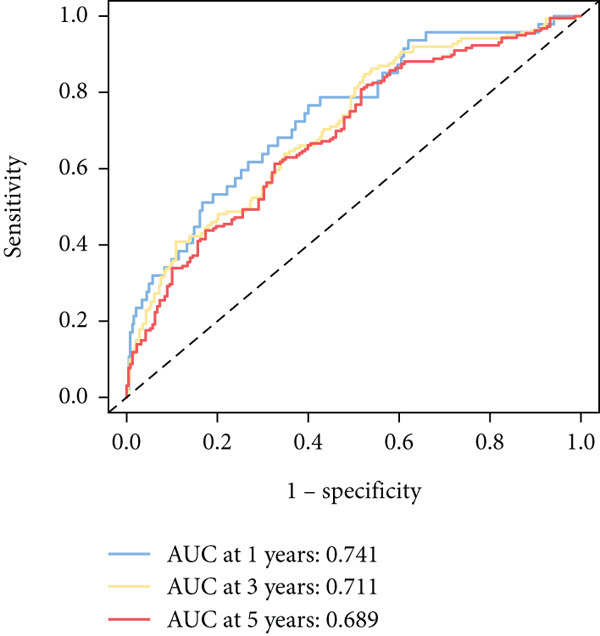
(m)

### 3.4. Evaluation of the ICAFBS Model

Next, we assessed the relationship between the distribution of patient survival status and risk scores [37], with five independent cohorts showing similar trends (Figure 5a). Subsequently, Cox regression methods were employed to estimate the independent prognostic value of ICAFBS. It was demonstrated that ICAFBS could serve as an excellent independent prognostic factor in LUAD (Figure 5b). Patients were further categorized into different subgroups based on their clinical characteristics, including age, stage, M, and N. Survival analysis demonstrated that our proposed ICAFBS still presented good performance in different subgroups (Figures 5c, 5d, 5e, and 5f). Moreover, it was found that the two groups differed significantly in terms of the stage, M and N. Patients in stage III‐IV, M0, and N1‐3 groups had greatly higher risk scores (Figure 5g). These results emphasize the impressive performance of ICAFBS.

Figure 5Evaluation of the ICAFBS model. (a) The distribution of the ICAFBS and clinical outcome of patients in each LUAD cohort. (b) Cox analysis confirms remarkable independent prognostic value of ICAFBS in LUAD. (c–f) The favorable power of ICAFBS in predicting patient outcomes in different subgroups (age, stage, M, and N) was detected by survival curves. (g) The comparison of ICAFBS score between different subgroups (stage, M, and N). ∗∗*p* < 0.01, ∗∗∗*p* < 0.001.

**Figure:**
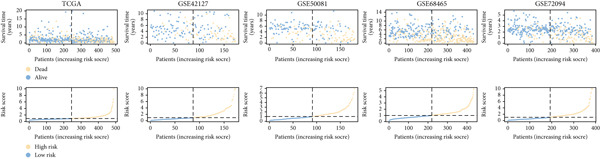
(a)

**Figure:**
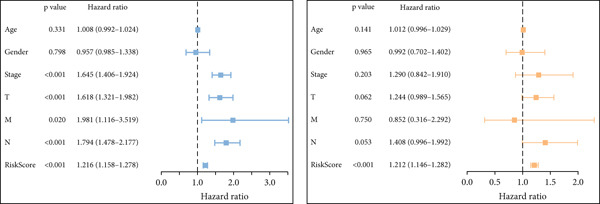
(b)

**Figure:**
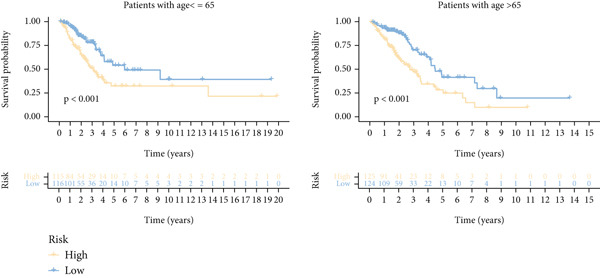
(c)

**Figure:**
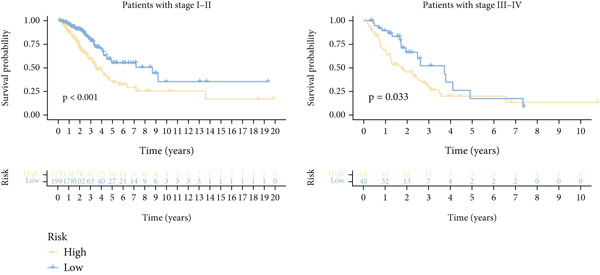
(d)

**Figure:**
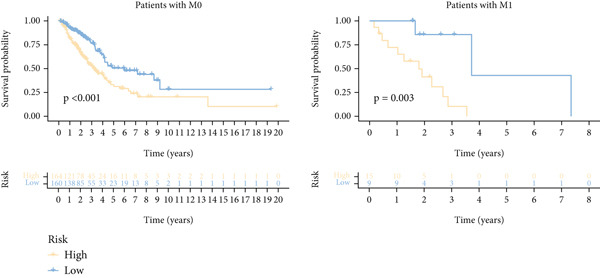
(e)

**Figure:**
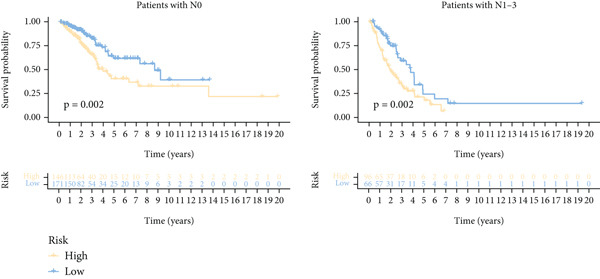
(f)

**Figure:**
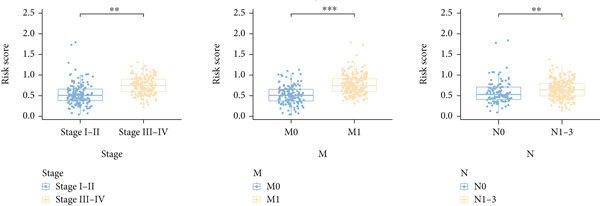
(g)

### 3.5. Correlation of Immune Landscape With ICAFBS

Based on different immunity algorithms, the relationship between two risk groups of immunocytes and ICAFBS was estimated. As indicated in Figure 5a, macrophages showed a positive, while T cells and NK cells displayed negative correlations with ICAFBS scores (Figure 6a). The results from ssGSEA analysis disclosed that the patients with high‐ICAFBS presented lower activity in the IFN2‐type response, HLA and T‐cell co‐inhibitory pathways, whereas the immune checkpoints as well as inflammation‐promoting pathways were remarkably activated (Figure 6b). It was demonstrated that high‐ICAFBS patients showed an increased expression pattern of checkpoints (Figure 6c). The inflammatory microenvironment also could serve a crucial effect in LUAD development, so we detected the association between ICAFBS and inflammatory factors. We found that high‐ICAFBS group exhibited an inflammatory activation state (Figure 6d). We then detected the performance of ICAFBS in the prediction of immune escape based on the TIDE database. The scores of TIDE, T cell dysfunction, and T cell exclusion were greatly higher in the high‐ICAFBS group, suggesting that these patients were prone to immune escape (Figures 6e, 6f, and 6g). In addition, besides TAM cells, pro‐cancer cells such as MDSC and CAF had higher scores in the high‐ICAFBS group (Figures 6h, 6i, and 6j).

Figure 6Correlation of immune landscape with ICAFBS. (a) The correlation between diverse immunocytes and ICAFBS. (b) Heatmap showing the difference in immune function pathways between the two groups. (c) The expression of immune checkpoints among the two groups. (d) The expression of inflammatory factor among the two groups. The association between diverse indicators of immune escape and ICAFBS, including (e) TIDE. (f) Dysfunction. (g) Exclusion. (h) MDSC. (i) CAF. (j) TAM. ∗∗*p* < 0.01, ∗∗∗*p* < 0.001, ns = not significant.

**Figure:**
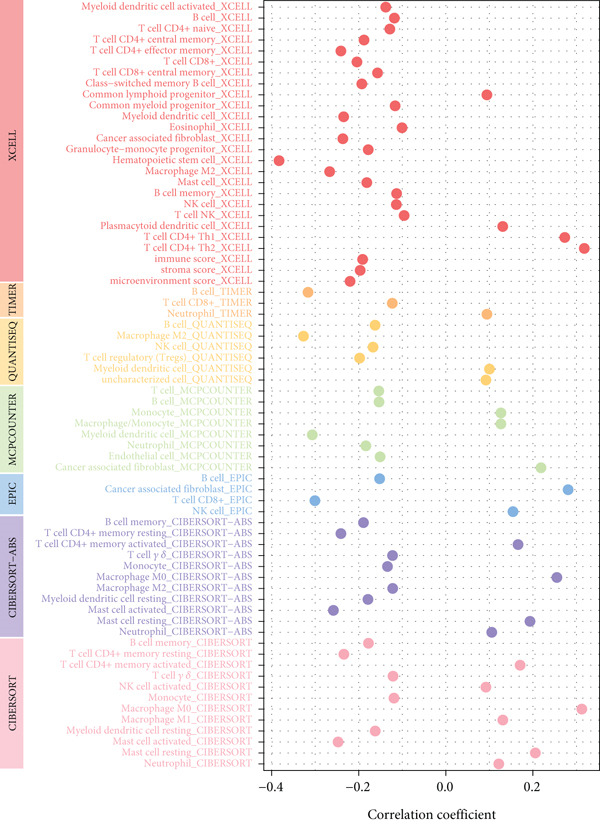
(a)

**Figure:**
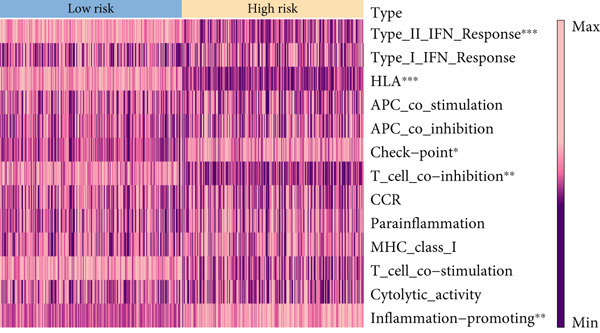
(b)

**Figure:**
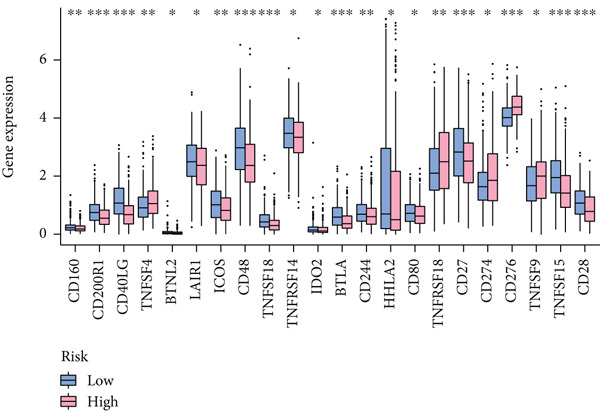
(c)

**Figure:**
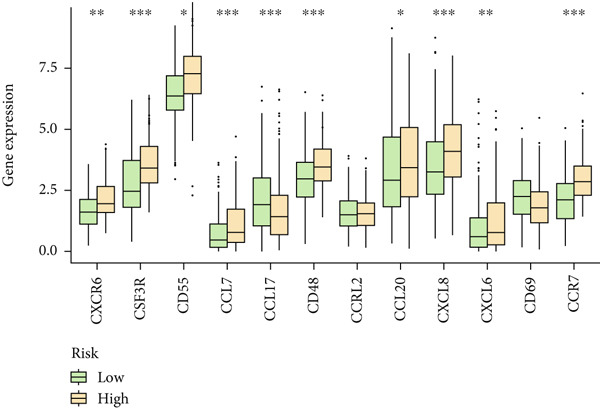
(d)

**Figure:**
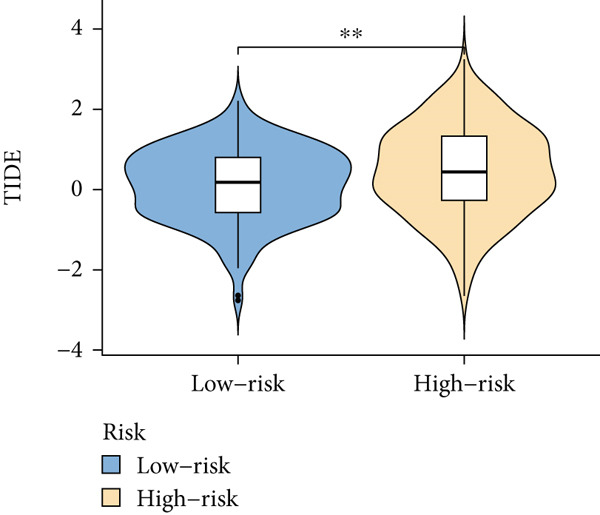
(e)

**Figure:**
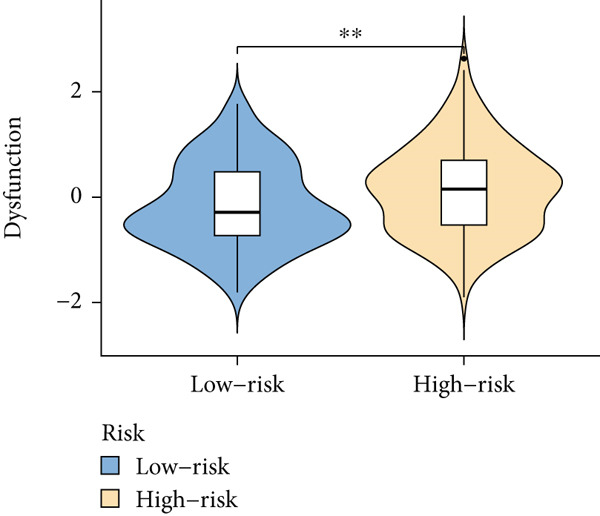
(f)

**Figure:**
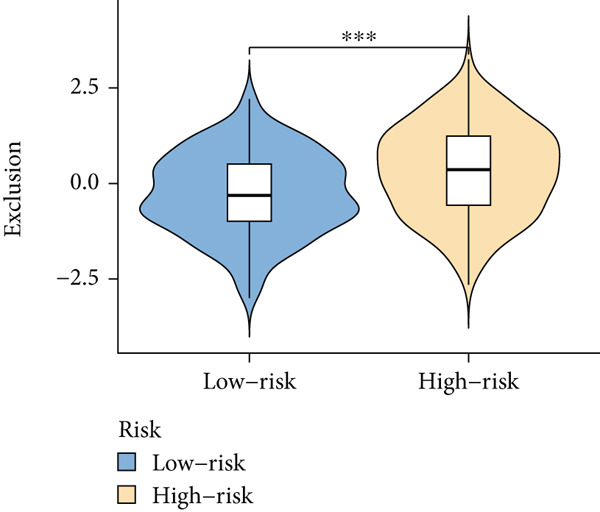
(g)

**Figure:**
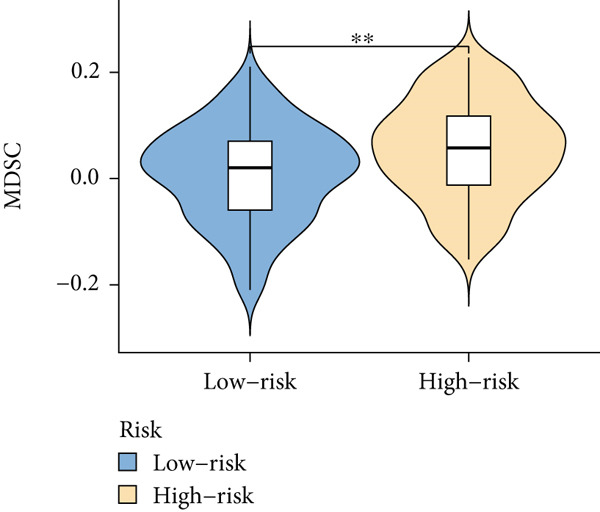
(h)

**Figure:**
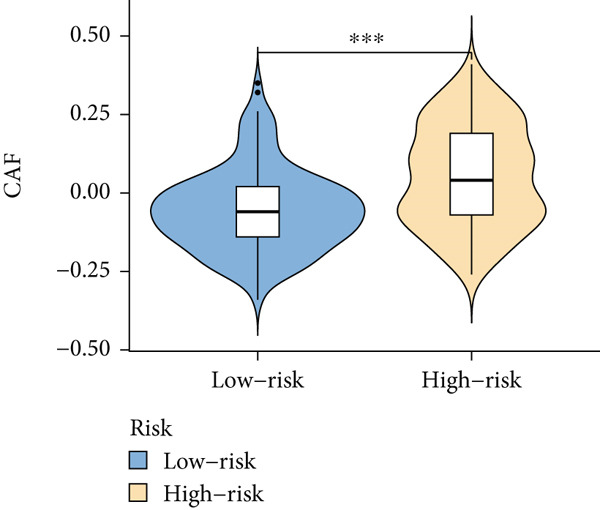
(i)

**Figure:**
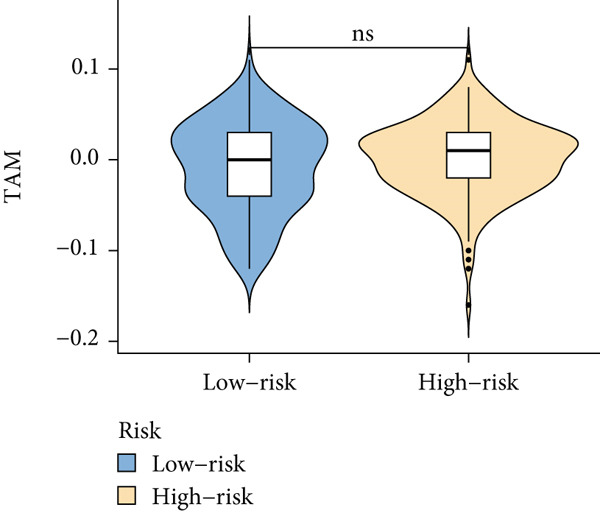
(j)

### 3.6. Drug Sensitivity Analysis

To detect the association between ICAFBS and drug sensitivity, the IC50 value of different drugs in LUAD samples was generated based on GDSC database. Notably, patients with high ICAFBS were sensitive to axitinib and P22077. In contrast, cisplatin, docetaxel, olaparib, rapamycin, sapitinib, and savolitinib were more effective in treating patients in the low ICAFBS group (Supplementary Figure 1).

### 3.7. Single‐Cell Landscape of ICAFBS in LUAD

To confirm our proposed ICAFBS at a single‐cell level, we first detected the four model genes in CAF population. It was indicated that these four hub genes were highly enriched in iCAF subtype by both UMAP plots and dot plots (Figure 7a,b). The results of the correlation analysis further demonstrated their positive correlation with classical cell markers of CAF (FAP, ACTA2, and PDGFRA), suggesting that they can serve as signature molecules reflecting iCAF population (Figure 7c). Based on the ICAFBS scoring system, we assigned a score to each cell and classified all cells into two risk subgroups based on the median value of the ICAFBS score (Figure 7d,e). Next, cell‐to‐cell communications were estimated by using CellChat to reflect cellular interactions between the two ICAFBS groups. It was observed that the high‐ICAFBS cells showed more intercellular interactions (Figure 7f). In the high‐ICAFBS group, tumors presented stronger cellular interactions with both fibroblasts, myeloid as well as endothelial cells (Figure 7g). Moreover, we found that ITGB2 and ICAM signaling were activated in the high‐ICAFBS group (Figures 7h, 7i, and 7j).

Figure 7Single‐cell landscape of ICAFBS in LUAD. (a) Distribution of the four genes in iCAF subtype. (b) Dot plot of the four genes of each CAF subtype. (c) Correlation analysis shows the positive correlation between the four genes and classical cell markers of CAF (FAP, ACTA2, and PDGFRA). (d) UMAP plot showing ICAFBS score for each cell. (e) UMAP plot of all cells colored by ICAFBS group. (f) Differences in cellular communication related to different ICAFBS scores. (g) Circos plots display cellular communication between major cell types from the two ICAFBS subgroups. (h) Overview of information flow differences between the two ICAFBS subgroups. (i,j) Circos plots reveal the ITGB2 and ICAM signaling pathway of related cells in the two ICAFBS subgroups.

**Figure:**
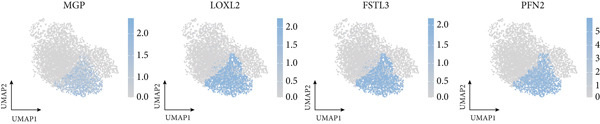
(a)

**Figure:**
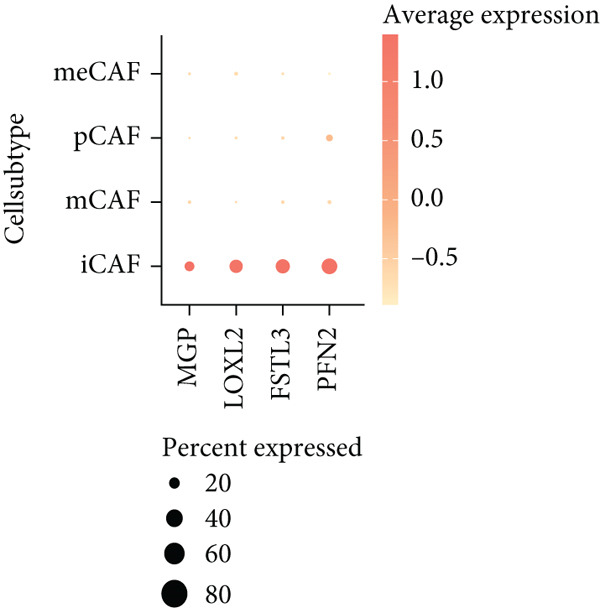
(b)

**Figure:**
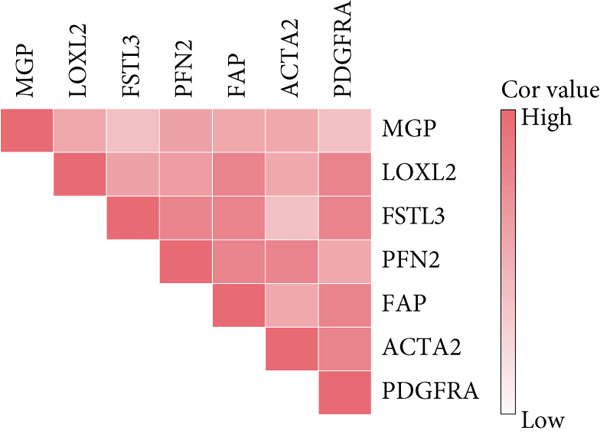
(c)

**Figure:**
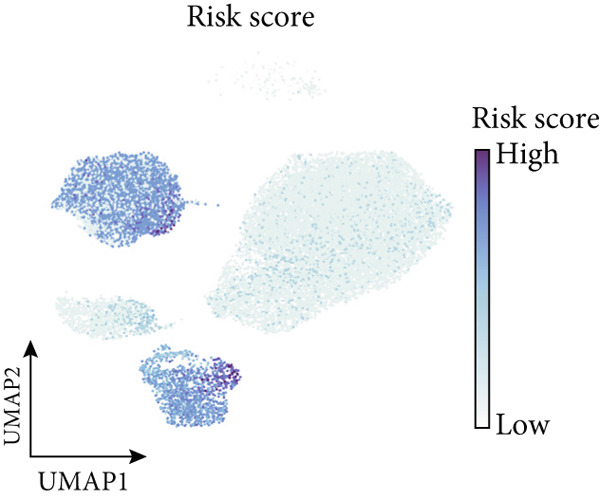
(d)

**Figure:**
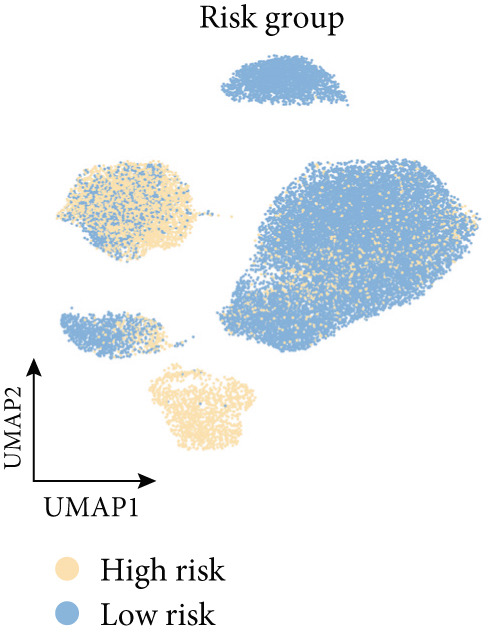
(e)

**Figure:**
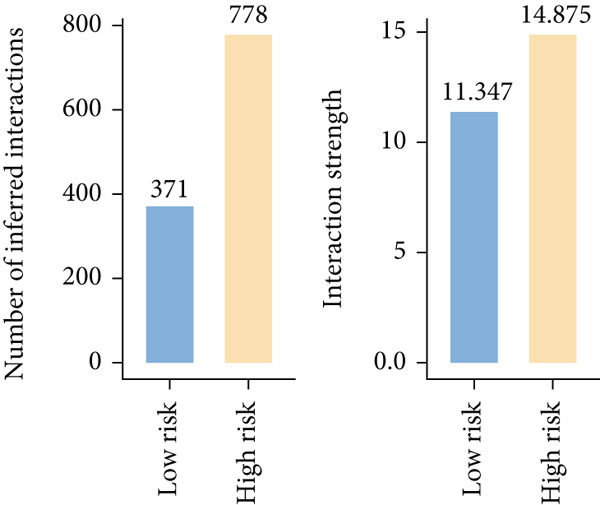
(f)

**Figure:**
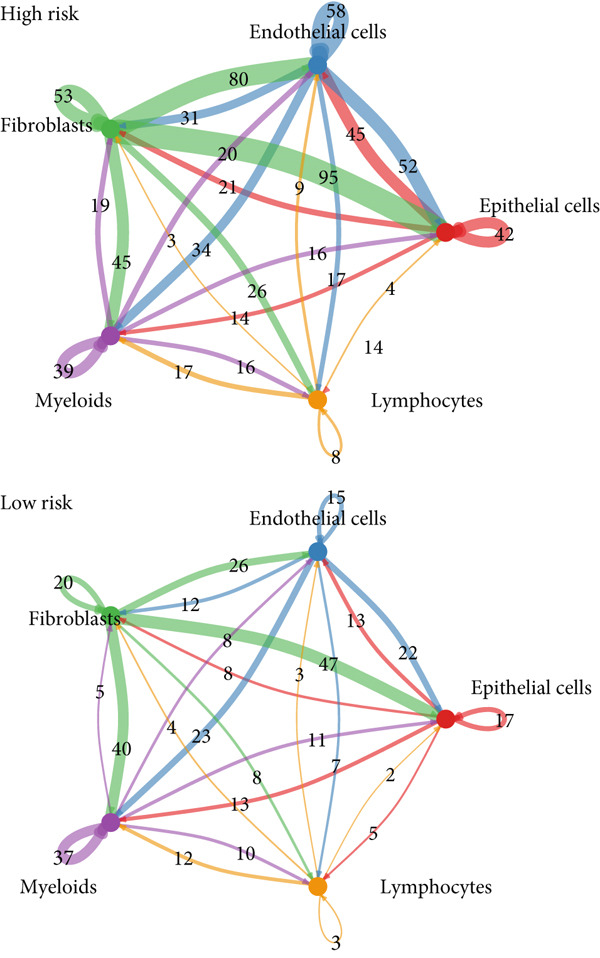
(g)

**Figure:**
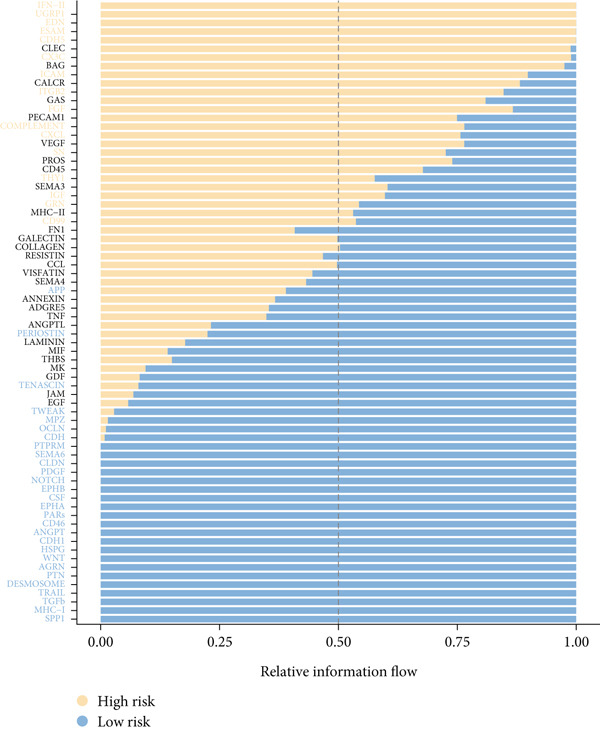
(h)

**Figure:**
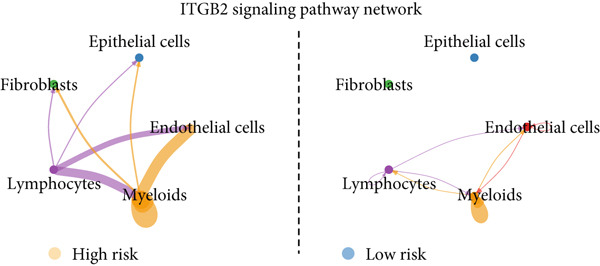
(i)

**Figure:**
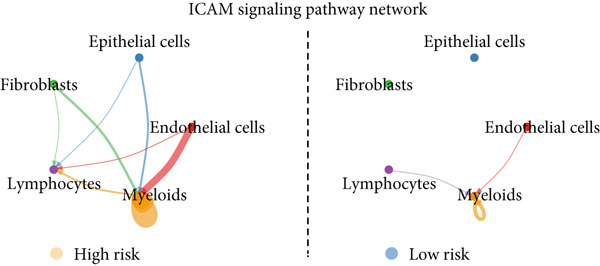
(j)

### 3.8. Verification of Key Features in LUAD Cells

Subsequently, we assessed the prognostic significance of four genes of ICAFBS in LUAD. Regarding OS, elevated MGP expression was markedly linked to favorable outcomes, whereas survival analysis indicated that the remaining three genes were strongly tied to adverse clinical results (Supplementary Figure 2A). Notably, patients exhibiting high FSTL3 expression showed poorer disease‐free survival (DFS) (Supplementary Figure 2B). Furthermore, we investigated FSTL3 and PFN2 expression patterns in LUAD tissue samples. IHC representative images from HPA database indicated that FSTL3 and PFN2 were highly expressed in tumor samples (Supplementary Figure 2C).

### 3.9. Determination of PFN2 as a Novel Oncogenic Player in LUAD

PFN2 was selected for functional validation due to its significant prognostic value and limited prior investigation in LUAD. To further validate the association between iCAFs and PFN2, we analyzed a publicly available spatial transcriptomics dataset. By applying the cell2location algorithm, using our single‐cell RNA‐seq data as a reference, we inferred the spatial distribution of iCAFs within LUAD tissue sections. The results demonstrated that areas enriched in iCAFs overlapped with regions of elevated PFN2 expression, suggesting a spatial co‐localization between iCAFs and PFN2 (Supplementary Figure 3). These findings support that PFN2 expression is strongly associated with iCAF populations within the tumor microenvironment (Supplementary Figure 3).

Next, we assessed the expression levels of PFN2 across various cell lines, revealing that PFN2 was remarkably upregulated in LUAD cells (Figure 8a). The transfection efficiency of sh‐PFN2 in A549 and H1975 cells was confirmed to be satisfactory (Figure 8b). As shown in Figure 8c, silencing PFN2 markedly suppressed cell viability through CCK‐8 assay. Furthermore, both clone formation and EdU staining demonstrated that PFN2 knockdown effectively inhibited the proliferation of LUAD cells (Figure 8d,e).

Figure 8Determination of PFN2 as a novel oncogenic player in LUAD. (a) Expression pattern of PFN2 in different cell lines was detected by qRT‐PCR assay. (b) PCR assay indicates the favorable transfection efficiency in LUAD cell lines. (c) Cell viability in different treatment groups was measured by CCK‐8 assay. (d,e) The role of PFN2 on proliferation in vitro was explored using (d) colony formation and (e) EdU assay. Scale bar = 50 *μ*m. ∗∗∗*p* < 0.001. Data are presented as mean ± SD from three independent biological replicates (*n* = 3).

**Figure:**
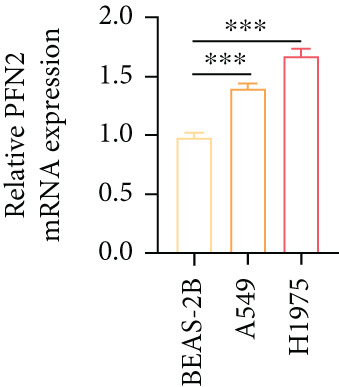
(a)

**Figure:**
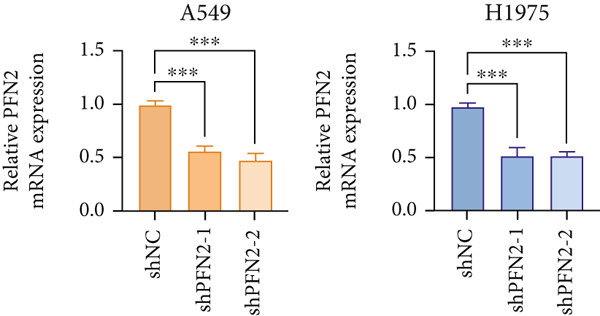
(b)

**Figure:**
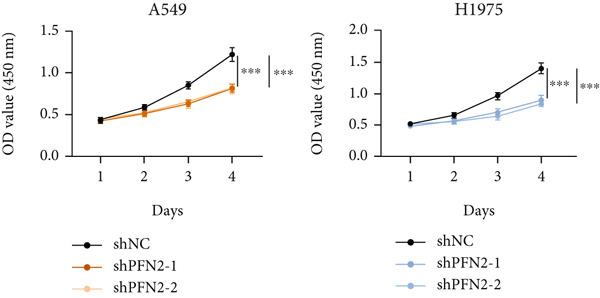
(c)

**Figure:**
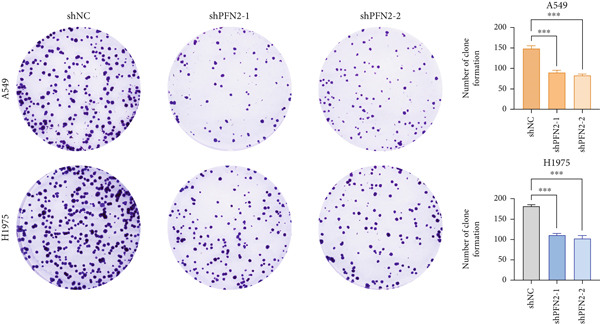
(d)

**Figure:**
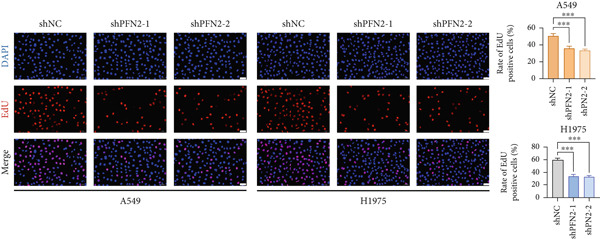
(e)

## 4. Discussion

Numerous studies have uncovered the critical function of CAF in tumorigenesis, development, and immune escape [25, 26, 38, 39]. Among CAF subsets, iCAFs are characterized by their high secretion of chemokines, modulating immune responses and promoting tumor‐supportive inflammation. The immunosuppressive TME driven by iCAFs could boost tumor growth and drug resistance [27, 28, 40]. Although the role of iCAFs has been explored in several malignancies, including breast and pancreatic cancers [41, 42], the specific relationship between iCAFs and LUAD remains largely underexplored. Notably, the current shortage of comprehensive research elucidating the molecular characteristics, prognostic significance, and functional roles of iCAFs in LUAD, highlighting an urgent need for further investigation in this field.

Accumulating evidence has shown that CAF‐related gene signatures are closely associated with prognosis and therapeutic responses across various cancer types, suggesting that CAFs are not merely passive bystanders but active drivers of tumor progression and drug resistance. For instance, in breast cancer, CAF‐derived IL‐6 has been implicated in enhancing tumor progression and radioresistance [43]. Similarly, in pancreatic ductal adenocarcinoma, iCAFs‐derived CXCL12 mediates immune exclusion by preventing T cell infiltration, contributing to immune checkpoint inhibitor resistance [44]. These studies highlight that targeting CAF subtypes, including iCAFs, may offer novel therapeutic opportunities. However, studies on CAFs in LUAD have largely neglected tumor heterogeneity, particularly the diverse functional subtypes of CAFs and their dynamic interactions with tumor and immune cells. Our proposed ICAFBS represents an important step in understanding the prognostic and potentially therapeutic significance of iCAFs in LUAD.

Through the robust machine learning approaches, we developed a four‐gene model (MGP, LOXL2, FSTL3, and PFN2) reflecting key biological processes associated with inflammatory CAF activity and tumor aggressiveness. Notably, the ICAFBS showed consistently favorable predictive performance in both the training cohort and multiple independent external datasets from GEO, demonstrating its strong generalizability. Survival analysis and AUC value indicate that ICAFBS was reliable in distinguishing patients at risk. Subgroup analysis further demonstrated that ICAFBS can also effectively stratify patients in different clinical subgroups, making it suitable for a wide range of LUAD patients. From a clinical perspective, this signature provides a practical tool for risk stratification, allowing clinicians to better estimate patient prognosis and personalize surveillance and treatment strategies. Moreover, by capturing stromal fibroblast‐driven tumor biology, ICAFBS adds a new dimension to current prognostic models that predominantly focus on tumor‐intrinsic genetic alterations, thus enhancing our capacity to predict LUAD outcomes in a more comprehensive and biologically informed manner. Moreover, the ICAFBS also provides important insights into the immune landscape and therapeutic responsiveness of LUAD, which holds significant implications for precision oncology. Our comprehensive immune profiling revealed that LUAD patients with high ICAFBS scores exhibited a markedly immunosuppressive tumor microenvironment, characterized by increased infiltration of TAMs, CAFs, and MDSCs, alongside decreased levels of cytotoxic T cells and NK cells. Additionally, patients with high ICAFBS score showed enhanced expression of multiple immune checkpoint molecules, including PD‐1, PD‐L1, and CTLA‐4, indicating a tumor milieu prone to immune evasion. The results indicated that the high ICAFBS group might experience diminished advantages from immune checkpoint blockade (ICB) monotherapy, attributable to an exceedingly suppressive immune environment.

In addition to immunotherapy, drug sensitivity analysis uncovered that high‐ICAFBS patients displayed differential sensitivity to several targeted agents and chemotherapeutic drugs. For example, patients with high ICAFBS scores were predicted to be more resistant to standard chemotherapies such as cisplatin and paclitaxel, yet potentially more responsive to agents targeting the stromal components or TGF‐*β* signaling pathways. These observations highlight the clinical utility of ICAFBS not only in prognostic risk assessment but also in guiding individualized treatment selection, including immunotherapy and targeted therapy combinations. By identifying patients with distinct immune and stromal features, our model offers a pathway for precision medicine in LUAD, facilitating the development of rational, patient‐tailored therapeutic strategies that may overcome current limitations in LUAD management.

Importantly, through integrative machine learning approaches, we identified four hub genes (MGP, LOXL2, FSTL3, and PFN2) that are closely associated with tumor development and progression. Matrix Gla protein (MGP) is well known for its role in extracellular matrix regulation and has been widely reported to be implicated in fibrotic processes in various tissues, including the dermal papilla and intestinal tract [45]. MGP has shown conflicting roles in cancer, including tumor suppression or promotion depending on the context [46]. As revealed by Li and his colleague, NF‐*κ*B pathway activation driven by MGP can contribute to colorectal cancer tumor growth and progression [47]. In contrast, Li demonstrated that LGR4 promotes breast cancer metastasis by downregulating MGP expression [48]. In our study, MGP was identified as a favorable prognostic marker in LUAD, which is consistent with findings by Caiado et al. [49]. LOXL2 (lysyl oxidase like 2), an enzyme involved in collagen crosslinking, is known to promote tumor invasiveness and metastasis through extracellular matrix remodeling and has been linked to poor outcomes in various tumors, including lung cancer [50]. LOXL2 was shown by Fan and colleagues to promote lung cancer metastasis and progression via CEBPA‐mediated upregulation, which inhibits BCL‐2 degradation [51]. In breast cancer, LOXL2‐activated PEAR1 phosphorylation facilitates metastasis by maintaining CD44 stability [52]. Similarly, it was shown that LOXL2^+^ iCAFs as greatly correlated with poor prognosis in bladder cancer through integrated analysis [53], further supporting our findings. FSTL3 (follistatin‐like 3), a secreted glycoprotein, was observed to boost tumor growth and immune evasion in multiple cancers [54, 55]. In colorectal cancer, FSTL3 promotes immune evasion by inhibiting c‐Myc degradation, ultimately contributing to the failure of immunotherapy [54]. Previous studies revealed that the upregulation of FSTL3, mediated by lncRNA DSCAM‐AS1, enhances the proliferation and metastasis of lung cancer cells [55, 56]. PFN2 (profilin 2), an actin‐binding protein, contributes to cytoskeletal reorganization, cell migration, and invasion. It was demonstrated by Guo and his colleague. to be transcriptionally activated by SIX2, thereby promoting stemness and progression in gastric cancer [57]. High PFN2 expression has also been linked to poor prognosis in breast cancer, where it facilitates metastasis through Smad2 upregulation [58, 59]. Collectively, these genes are not only reflective of iCAF biological activity but also intimately involved in the malignant behavior of LUAD cells, supporting the robust prognostic value of our ICAFBS and providing potential targets for future therapeutic interventions.

Beyond its prognostic and immunological insights, ICAFBS holds promising potential as a companion diagnostic tool in the clinical setting. Given its strong association with immune suppression and therapeutic resistance, ICAFBS could be utilized to stratify LUAD patients prior to treatment, aiding clinicians in identifying individuals who may benefit less from conventional immunotherapies and may require combinatory or alternative therapeutic strategies. Notably, as CAF‐targeted therapies are increasingly being developed, such as inhibitors of TGF‐*β* signaling, FAP, or IL‐6/STAT3 pathway modulators [60–64], the ICAFBS could serve as a critical tool to guide treatment decisions. By identifying patients with high ICAFBS scores, oncologists could consider integrating CAF‐directed agents into their treatment regimens to overcome stromal‐mediated resistance. Furthermore, ICAFBS may assist in monitoring therapeutic response or disease progression, functioning as a dynamic biomarker reflective of the stromal landscape. For instance, patients receiving CAF‐targeted interventions could be longitudinally assessed using ICAFBS to evaluate whether stromal remodeling or immune activation is occurring, thereby optimizing therapeutic adjustments in real time. Future clinical trials incorporating ICAFBS into patient selection criteria would be valuable in validating its utility as a stratification marker and in personalizing LUAD management based on tumor–stroma interactions.

This study has several limitations. First, it is based on retrospective public datasets, which may introduce selection bias and limit generalizability [65]. Although multiple external cohorts were used for validation, prospective clinical studies are needed to further confirm the robustness of ICAFBS. Second, experimental validation was limited to in vitro assays using LUAD cell lines. The lack of patient‐derived samples or in vivo models restricts the interpretation of the findings in the context of the native tumor microenvironment. Future studies involving clinical specimens and animal models will be essential to validate the clinical utility of ICAFBS.

## 5. Conclusion

This study reveals the significant role of iCAFs in LUAD and develops a prognostic model (ICAFBS) based on their characteristic genes. The ICAFBS not only effectively predicts patient survival outcomes but is also closely related to the immune landscape, immune evasion, and treatment response, providing new insights for the prognosis assessment and personalized treatment of LUAD patients.

## Conflicts of Interest

The authors declare no conflicts of interest.

## Author Contributions

Xiaoyue Zhou and Dan Yang designed the original study. Xiaoyue Zhou, Cong Fu, Ying Fu, and Ting Jiao collected analyzed the data. Chenyu Zhao conducted experimental work. Xiaoyue Zhou and Dan Yang drafted and revised the manuscript. All authors contributed to the revision of this manuscript.

## Funding

This study is supported by the Changzhou Science and Technology Plan Project (Applied Basic Research Special Project), CJ20245037.

## Supporting information


**Supporting Information** Additional supporting information can be found online in the Supporting Information section. Supplementary Table 1: Summary of machine learning algorithms and implementation details. Supplementary Table 2: Knockdown sequences of PFN2. Supplementary Table 3: The primer sequences of each gene in this study. Supplementary Table 4: The marker genes in each CAF subtype. Supplementary Table 5: A total of 56 prognostic genes identified by univariate cox analysis. Supplementary Table 6: Multivariate cox analysis determined four hub genes for construction of predictive model. Supplementary Figure 1: Drug sensitivity analysis. Supplementary Figure 2: Verification of key features in LUAD. Supplementary Figure 3: Spatial transcriptomics analysis.

## Data Availability

The public datasets to support the results of this study can be obtained from TCGA (https://portal.gdc.cancer.gov/), GEO (https://www.ncbi.nlm.nih.gov/geo/), and EMBL‐EBI databases (https://www.ebi.ac.uk/).
