# Supplementary material for: A Multi‐Faceted Approach to Explore the Role of Inflammatory CAFs, Providing Prognostic Value and Therapeutic Implications in Lung Adenocarcinoma
Source: Int J Genomics. 2025 Dec 12;2025:6004629. doi: 10.1155/ijog/6004629 (PMC12701138; doi:10.1155/ijog/6004629)
Supplement: Supplementary file 1 — Supporting Information Additional supporting information can be found online in the Supporting Information section. Supplementary Table 1: Summary of machine learning algorithms and implementation details. Supplementary Table 2: Knockdown sequences of PFN2. Supplementary Table 3: The primer sequences of each gene in this study. Supplementary Table 4: The marker genes in each CAF subtype. Supplementary Table 5: A total of 56 prognostic genes identified by univariate cox analysis. Supplementary Table 6: Multivariate cox analysis determined four hub genes for construction of predictive model. Supplementary Figure 1: Drug sensitivity analysis. Supplementary Figure 2: Verification of key features in LUAD. Supplementary Figure 3: Spatial transcriptomics analysis. [file IJOG-2025-6004629-s001.docx]

**Supplementary Materials**

**Supplemental Method**

**Development of iCAF-based signature (ICAFBS)**

The RSF model was implemented using the randomForestSRC v3.2.3 package. Two key hyperparameters were tuned: ntree (number of trees in the forest) and mtry (number of variables randomly selected for splitting at each node). We performed a grid search across all possible (ntree, mtry) pairs under a Leave-One-Out Cross Validation (LOOCV) framework and selected the combination that yielded the highest concordance index (C-index).

The survival-SVM model was implemented using the survivalsvm package. We employed the “ranking” type survival SVM, which is suitable for learning from censored data. We tested different kernel types including linear, polynomial, and radial basis function (RBF) kernels. Hyperparameters such as kernel type and cost parameter were optimized using grid search within the LOOCV framework, and the configuration achieving the best C-index was selected.

Enet, Lasso, and Ridge regression models were constructed using the glmnet v4.1-8 package. The regularization parameter (λ) was optimized via LOOCV, and the α parameter, controlling the L1-L2 penalty ratio, was evaluated over the interval from 0 to 1 in increments of 0.1. The stepwise Cox regression model was implemented through the survival package using the Akaike Information Criterion (AIC) as the selection metric. Directional search modes included both forward and backward steps.

CoxBoost was applied using the CoxBoost package, which supports Cox proportional hazards modeling through componentwise likelihood-based boosting. LOOCV was used to determine the optimal penalty value via the optimCoxBoostPenalty function, and the number of boosting iterations was tuned using the cv.CoxBoost function. The final dimensionality of the multivariate Cox model was established based on the principal routine from the CoxBoost framework.

The plsRcox model was fitted using the plsRcox package, where the cv.plsRcox function identified the optimal number of components, and the plsRcox function applied partial least squares regression in a generalized linear model context. SuperPC modeling was performed using the superpc package, which extends principal component analysis by generating supervised linear combinations of features that maximize variance. The superpc.cv function employed LOOCV to determine the optimal threshold for feature inclusion. To address the challenge of small validation datasets, a pre-validation strategy was utilized.

The GBM model, implemented using the superpc package, employed the cv.gbm function to identify the number of trees that minimize cross-validation error. Model fitting was carried out using the gbm function for generalized boosted regression.

**Supplementary Tables**

**Supplementary Table 1. Summary of machine learning algorithms and implementation details.**

| **Algorithm** | **Key Hyperparameters** | **Tuning Method** | **R Package** | **Version** |
| --- | --- | --- | --- | --- |
| Random Survival Forest (RSF) | ntree, mtry | Grid search + LOOCV | randomForestSRC | v3.2.3 |
| Elastic Net (Enet) | alpha (0-1), lambda | Grid search + LOOCV | glmnet | v4.1-8 |
| Lasso | lambda | LOOCV | glmnet | v4.1-8 |
| Ridge | lambda | LOOCV | glmnet | v4.1-8 |
| Stepwise Cox | Direction (forward, backward), Criterion (AIC) | Stepwise search | survival | v3.5-7 |
| CoxBoost | penalty, stepno | optimCoxBoostPenalty, cv.CoxBoost | CoxBoost | v1.4 |
| Partial Least Squares Cox (plsRcox) | number of components | cv.plsRcox | plsRcox | v1.7.1 |
| Supervised Principal Components (SuperPC) | threshold for feature inclusion | superpc.cv + Pre-validation | superpc | v1.12 |
| Generalized Boosted Model (GBM) | n.trees, interaction.depth, shrinkage | cv.gbm | gbm | v2.1.8 |
| Survival Support Vector Machine (SVM) | kernel (linear, polynomial, RBF), cost | Grid search + LOOCV | survivalsvm | v0.1-2 |

**Supplementary Table 2. Knockdown sequences of PFN2.**

| **Gene** | **Sequence** | |
| --- | --- | --- |
| sh-PFN2-1 |  | 5′-GCTGGTAGAGTCTTGGTCTTT-3′ |
| sh-PFN2-2 |  | 5′-ACAATGGACATCCGGACAAAG-3′ |

**Supplementary Table 3. The primer sequences of each gene in this study.**

| **Gene** | **Sequence** | |
| --- | --- | --- |
| PFN2 | Forward | 5′-ATGATTGTAGGAAAAGACCGGGA-3′ |
|  | Reverse | 5′-GCAGTCACCATCGACGTATAGAC-3′ |
| GAPDH | Forward | 5′-GTCTCCTCTGACTTCAACAGCG-3′ |
|  | Reverse | 5′-ACCACCCTGTTGCTGTAGTAGCCAA-3′ |

**Supplementary Table 4. The marker genes in each CAF subtype.**

| Cluster | Gene |
| --- | --- |
| iCAF | CFD |
| iCAF | MGP |
| iCAF | CXCL12 |
| iCAF | CXCL14 |
| iCAF | CCL11 |
| iCAF | APOD |
| iCAF | C7 |
| iCAF | IGF1 |
| iCAF | C3 |
| iCAF | ITM2A |
| mCAF | COL1A1 |
| mCAF | MMP11 |
| mCAF | CTHRC1 |
| mCAF | COL11A1 |
| mCAF | POSTN |
| mCAF | COL1A2 |
| mCAF | COL3A1 |
| mCAF | FN1 |
| mCAF | COL10A1 |
| mCAF | VCAN |
| pCAF | TOP2A |
| pCAF | IGFBP2 |
| pCAF | SCG2 |
| pCAF | STMN1 |
| pCAF | CENPF |
| pCAF | NUSAP1 |
| pCAF | PLAU |
| pCAF | PTTG1 |
| pCAF | TUBA1B |
| pCAF | IFI27 |
| meCAF | NDRG1 |
| meCAF | BNIP3 |
| meCAF | ADM |
| meCAF | SLC2A1 |
| meCAF | ENO1 |
| meCAF | VEGFA |
| meCAF | HILPDA |
| meCAF | ANGPTL4 |
| meCAF | TMEM158 |
| meCAF | MMP1 |

**Supplementary Table 5. A total of 56 prognostic genes identified by univariate cox analysis.**

| **Gene** | **HR** | **HR.95L** | **HR.95H** | **Pvalue** |
| --- | --- | --- | --- | --- |
| ITGB1 | 1.521873 | 1.209621 | 1.914731 | 0.000338 |
| PVR | 1.49531 | 1.178665 | 1.89702 | 0.00092 |
| PLOD1 | 1.445481 | 1.117286 | 1.87008 | 0.005048 |
| FBN2 | 1.441952 | 1.219054 | 1.705606 | 1.94E-05 |
| VEGFC | 1.407553 | 1.219226 | 1.624968 | 3.09E-06 |
| OXTR | 1.391788 | 1.118087 | 1.73249 | 0.003086 |
| LOXL2 | 1.354193 | 1.186355 | 1.545776 | 7.08E-06 |
| MSX1 | 1.327915 | 1.060382 | 1.662947 | 0.013484 |
| FSTL3 | 1.316076 | 1.115198 | 1.553137 | 0.001153 |
| LAMC1 | 1.313939 | 1.037872 | 1.663437 | 0.023276 |
| COLGALT1 | 1.298241 | 1.020636 | 1.651353 | 0.033473 |
| ITGA5 | 1.294507 | 1.099704 | 1.523818 | 0.001921 |
| BMP1 | 1.288285 | 1.03683 | 1.600723 | 0.02223 |
| SERPINH1 | 1.284104 | 1.049727 | 1.570812 | 0.015018 |
| CALU | 1.282869 | 1.035448 | 1.589413 | 0.022691 |
| SNAI2 | 1.274124 | 1.114301 | 1.45687 | 0.000396 |
| MMP14 | 1.268753 | 1.078475 | 1.492601 | 0.004088 |
| GPC1 | 1.261815 | 1.078059 | 1.476894 | 0.00378 |
| NID2 | 1.26007 | 1.077238 | 1.473933 | 0.003851 |
| DKK1 | 1.242586 | 1.156396 | 1.3352 | 3.19E-09 |
| PMEPA1 | 1.240168 | 1.084998 | 1.41753 | 0.001599 |
| PLOD2 | 1.239912 | 1.09428 | 1.404925 | 0.000743 |
| PLAUR | 1.237757 | 1.063858 | 1.440082 | 0.005757 |
| LAMC2 | 1.234737 | 1.108357 | 1.375528 | 0.00013 |
| SPOCK1 | 1.221852 | 1.086407 | 1.374184 | 0.00083 |
| ADAM12 | 1.220787 | 1.047468 | 1.422785 | 0.010662 |
| MCM7 | 1.214344 | 1.007053 | 1.464303 | 0.041996 |
| LOX | 1.210777 | 1.049034 | 1.397458 | 0.008942 |
| SLC6A8 | 1.194076 | 1.052013 | 1.355323 | 0.006059 |
| COL4A1 | 1.193793 | 1.016057 | 1.402619 | 0.031269 |
| COL7A1 | 1.181684 | 1.050237 | 1.329582 | 0.005526 |
| COL4A2 | 1.181203 | 1.002287 | 1.392056 | 0.046898 |
| SERPINE1 | 1.164233 | 1.038193 | 1.305575 | 0.009292 |
| LAMA3 | 1.162382 | 1.053763 | 1.282198 | 0.002645 |
| COL5A2 | 1.158668 | 1.020677 | 1.315314 | 0.022828 |
| CDH2 | 1.152226 | 1.011054 | 1.31311 | 0.033602 |
| MFAP5 | 1.147996 | 1.003803 | 1.312902 | 0.043863 |
| IGFBP3 | 1.147283 | 1.01652 | 1.294867 | 0.026058 |
| COL5A1 | 1.145955 | 1.007876 | 1.302951 | 0.037551 |
| POSTN | 1.144774 | 1.017675 | 1.287747 | 0.024337 |
| COL1A2 | 1.128997 | 1.000263 | 1.274298 | 0.049504 |
| COL1A1 | 1.119576 | 1.005831 | 1.246184 | 0.038798 |
| NT5E | 1.114957 | 1.013532 | 1.22653 | 0.025339 |
| COL11A1 | 1.087326 | 1.010601 | 1.169877 | 0.024935 |
| ELN | 0.880129 | 0.784177 | 0.987821 | 0.030158 |
| DCN | 0.867426 | 0.762513 | 0.986774 | 0.030587 |
| CADM1 | 0.844735 | 0.741265 | 0.962647 | 0.011374 |
| MGP | 0.815289 | 0.727037 | 0.914254 | 0.000477 |
| FBLN5 | 0.809156 | 0.684646 | 0.956309 | 0.012992 |
| ID2 | 0.790734 | 0.647189 | 0.966117 | 0.021606 |
| LAMA2 | 0.784856 | 0.65016 | 0.947458 | 0.011676 |
| ABI3BP | 0.783611 | 0.653218 | 0.940033 | 0.008641 |
| SLIT3 | 0.744747 | 0.603787 | 0.918614 | 0.005907 |
| SNTB1 | 0.742381 | 0.625094 | 0.881675 | 0.000686 |
| FUCA1 | 0.699725 | 0.574588 | 0.852116 | 0.000383 |
| SGCG | 0.555918 | 0.356586 | 0.866678 | 0.009555 |

**Supplementary Table 6. Multivariate cox analysis determined four hub genes for construction of predictive model.**

| **Gene** | **Coefficient** | **HR** | **HR.95L** | **HR.95H** | **Pvalue** |
| --- | --- | --- | --- | --- | --- |
| MGP | -0.233 | 0.837039 | 0.73306 | 0.955768 | 0.008578 |
| LOXL2 | 0.154 | 1.320158 | 1.149609 | 1.516008 | 8.31E-05 |
| FSTL3 | 0.207 | 1.58809 | 1.101611 | 1.311357 | 7.83E-06 |
| PFN2 | 0.237 | 2.253553 | 1.956759 | 2.486995 | 0.009499 |

**Supplementary Figures**

**Supplementary Figure 1. Drug sensitivity analysis.**


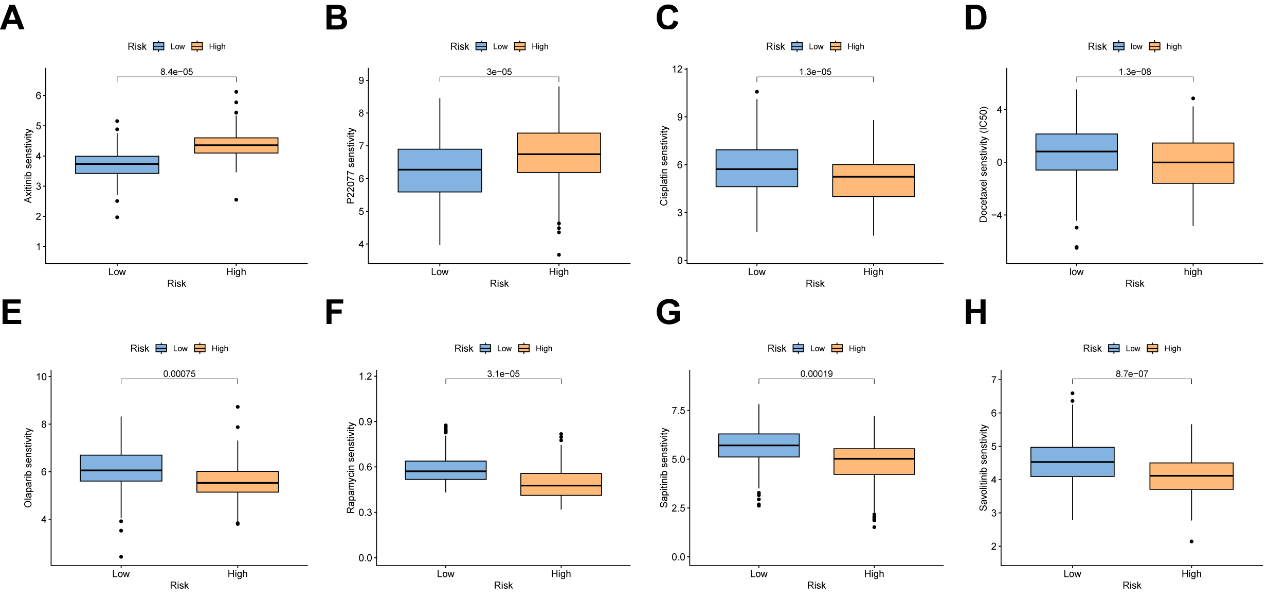


**Supplementary Figure 1.** The comparison of the sensitivity to different drugs between two ICAFBS subgroups, including (A) Axitinib. (B) P22077. (C) Cisplatin. (D) Docetaxel. (E) Olaparib. (F) Rapamycin. (G) Sapitinib. (H) Savolitinib.

**Supplementary Figure 2. Verification of key features in LUAD.**


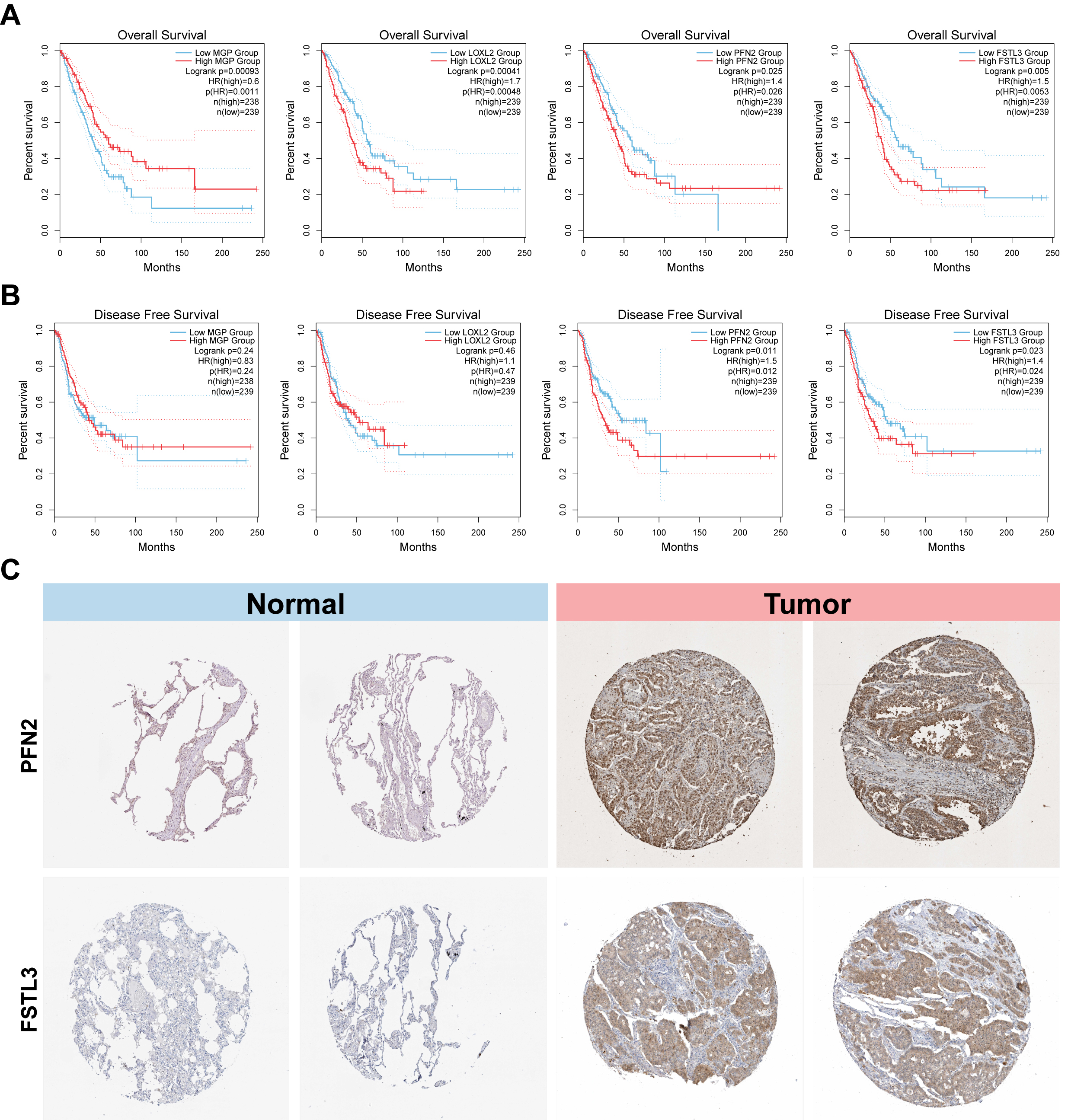


(A) Survival analysis demonstrates the difference in OS of four key genes between in two groups of expression levels.

(B) Survival analysis demonstrates the difference in DFS of four key genes between in two groups of expression levels.

(C) immunohistochemical (IHC) assays show the expression pattern of PFN2 and FSTL3.

**Supplementary Figure 3. Spatial transcriptomics analysis.**


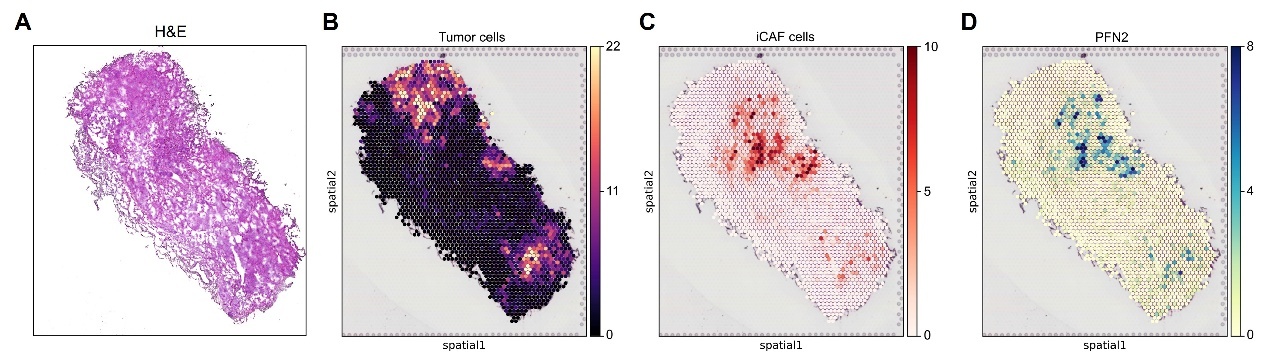


Representative LUAD tissue section showing (A) hematoxylin and eosin (H&E) staining, (B) spatial distribution of tumor cells, (C) inferred iCAF localization based on cell2location deconvolution using scRNA-seq reference profiles, and (D) spatial expression of PFN2.
